# The critical role of BMP signaling in gastric epithelial cell differentiation revealed by organoids

**DOI:** 10.1186/s13619-025-00237-x

**Published:** 2025-05-16

**Authors:** Fan Hong, Xiaodan Wang, Nanshan Zhong, Ze Zhang, Shibo Lin, Mengxian Zhang, Haonan Li, Yuan Liu, Yalong Wang, Lianzheng Zhao, Xiao Yang, Hongwen Zhou, Hui Liang, Ye-Guang Chen

**Affiliations:** 1 Guangzhou National Laboratory, Guangzhou, 510005 China; 2 The State Key Laboratory of Membrane Biology, Tsinghua-Peking Center for Life Sciences, School of Life Sciences, Tsinghua University, Beijing, 100084 China; 3 The MOE Basic Research and Innovation Center for the Targeted Therapeutics of Solid Tumors, School of Basic Medical Sciences, Jiangxi Medical College, Nanchang University, Nanchang, 330031 China; 4 Department of General Surgery, The First Affiliated Hospital of Nanjing Medical University, Nanjing, 210029 China; 5 State Key Laboratory of Proteomics, National Center for Protein Sciences (Beijing), Beijing Proteome Research Center, Beijing Institute of Lifeomics, Beijing, 102206 China; 6 Department of Endocrinology and Metabolism, The First Affiliated Hospital of Nanjing Medical University, Nanjing, 210029 China; 7 State Key Laboratory of Trauma, Burns and Combined Injury, Department of Shock and Transfusion, Research Institute of Surgery, Daping Hospital, Army Medical University, Chongqing, 400042 China

**Keywords:** Niche factors, Gastric epithelium, Cell differentiation, Organoids, Single cell RNA-seq

## Abstract

The efficient differentiation of adult gastric stem cells into specific epithelial cell types is crucial for gastric homeostasis. Although it is well appreciated that the niche plays a critical role in gastric epithelium cell differentiation, the relevant molecular factors and the underlying regulatory mechanisms remain poorly understood. In this study, by combining the knowledge of the niche cells obtained from single-cell RNA sequencing and manipulation of signaling pathways, we achieved effective differentiation of various gastric epithelial cell types in mouse and human gastric organoids. These in vitro differentiated cells showed a similar gene expression profile to those in gastric tissues. Specifically, BMP4 signaling stimulates pit cell and parietal cell differentiation. Furthermore, BMP4 and EGF signaling cooperate to enhance pit cell differentiation, whereas inhibition of TGF-β and BMP4 signaling promotes chief cell differentiation. We demonstrated that Zbtb7b is a novel regulator controlling pit cell differentiation. In addition, BMP4, together with the small molecule Isoxazole 9, promotes parietal and enteroendocrine cell differentiation. Our data also revealed the different requirements of parietal and chief cell differentiation between mouse and human. Together, our findings provide a mechanistic insight into gastric epithelial cell differentiation and uncover its similarities and differences between mouse and human, laying a foundation for future investigation and potential clinical use of gastric organoids.

**Supplementary Information:**

The online version contains supplementary material available at 10.1186/s13619-025-00237-x.

## Background

**Fig. 1:**
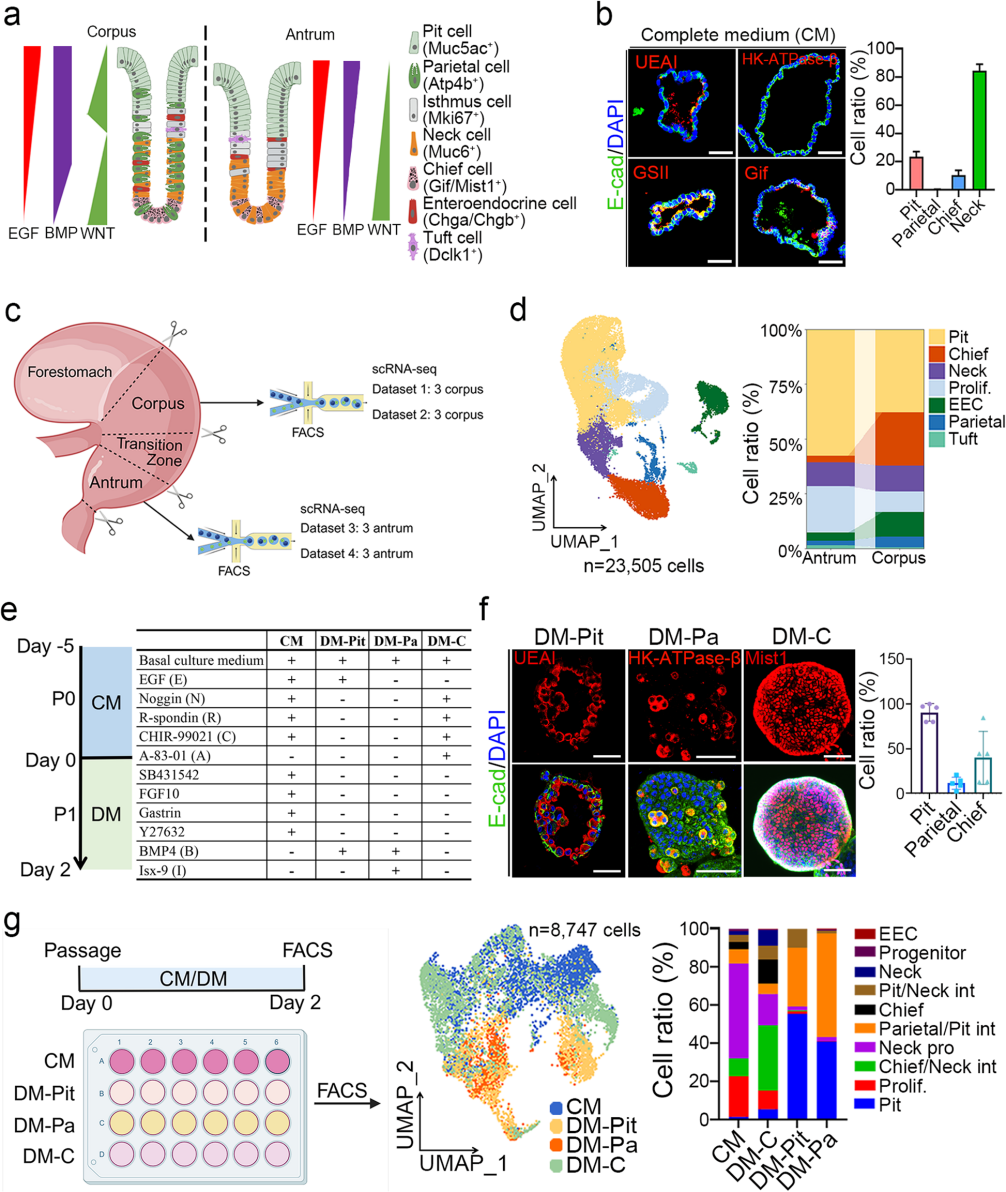
Establishment of mouse gastric organoids for cell differentiation studies. **a** A schematic depicting the cell landscape of a mouse gastric gland in the corpus and antrum and signaling pathway gradients. **b** Immunofluorescent staining of UEAI, HK-ATPase-β, Gif, GSII and E-cad, and quantification in mouse gastric organoids cultured in complete medium (CM) (*n* = 3). Scale bar: 50 μm. **c** A schematic illustration of 4 scRNA-seq datasets derived from mouse stomachs, including the corpus and antrum. **d** UMAP visualization of 7 single-cell clusters comprising 23,505 cells from mouse stomach epithelial tissues, along with corresponding cell ratio (right panel). **e** Differentiation process (left panel) and a comparison of medium composition between the complete medium (CM) and the mouse gastric epithelial cell differentiation medium for pit cell (DM-Pit), parietal cell (DM-Pa) and chief cell (DM-C). The detailed component for basal culture medium and the concentrations of these growth factors or molecules are listed in Table S1. **f** Immunofluorescent staining of UEAI, HK-ATPase-β, Mist1 and E-cad, and quantification in mouse gastric organoids cultured under the indicated conditions (*n* = 5). Scale bars, 20 μm. **g** Mouse gastric organoids cultured under different media for 2 days (left panel), followed by single-cell sorting for scRNA-seq of these organoids. UMAP visualization of integrated mouse gastric organoids cultured under the indicated conditions (*n* = 4) (middle panel), along with corresponding cell ratio (right panel). EEC: enteroendocrine cell; Pit/Neck int: pit and neck intermediate; Parietal/Pit int: parietal and pit intermediate; Neck pro: neck progenitor; Chief/Neck int: chief and neck intermediate; Prolif.: Proliferative cells. All immunofluorescence images were counter-stained with DAPI to show nuclei

The stomach is involved in food digestion, hormone secretion and defense against foodborne microbial pathogens. In the gastric glands, stem cells possess the ability to differentiate into various types of gastric epithelial cells (Barker et al. 2010). These include mucus-secreting cells (pit cell and neck mucus cell), acid-secreting cells (parietal cell), pepsinogen-secreting cells (chief cell), and enteroendocrine cells. The self-renewal and differentiation processes of the gastric epithelial cells are critical for maintaining gastric homeostasis and regulated by signaling networks between epithelial and mesenchymal cells. Wnt signaling plays a crucial role in maintaining the stemness and proliferative capacity of gastric progenitor cells (Barker et al. 2010; Leushacke et al. 2017). Epidermal growth factor (EGF) ligands, such as transforming growth factor-α (TGFα), which are concentrated at the upper part of the gastric gland, promote pit cell differentiation (Takada et al. 2023; Wolffling et al. 2021). Bone morphogenetic protein 4 (BMP4) signaling, which is most prominent in the gastric isthmus, governs the localization and differentiation of parietal cells (Shinohara et al. 2010; Wolffling et al. 2021). The gradients of BMP4, EGF, and Wnt signaling collectively shape the cell lineage landscape (Bartfeld and Koo 2017; Sigal et al. 2017; Wolffling et al. 2021) **(**Fig. 1a**)**. Chemical molecules that modulate signaling pathways can also regulate gastric epithelial cell differentiation. For instance, metformin promotes parietal cell maturation in mice (Miao et al. 2020). Despite these studies, how signaling pathways control differentiation of gastric epithelial cells is largely unclear.

Organoids are a good model to investigate the molecular mechanisms underlying cell renewal and differentiation. Lgr5-positive cells in the mouse antrum has been shown to be capable of generating various gastric epithelial cells and forming organoids in vitro (Barker et al. 2010). What are the stem cells in the corpus are still under debate although various cells have been suggested to fulfill the stem cell function, including the cells expressing Lgr5, Mist1, Troy, p57kip2, Stmn1, or Lrig1 (Han et al. 2019; Huh et al. 2010; Lee et al. 2022; Nienhuser et al. 2021; Stange et al. 2013). Two-dimension culture has also been used for cell differentiation from isthmus-like stem cells(Huebner et al. 2023). Although complex three-layered human gastric organoids could be derived from pluripotent stem cells (Eicher et al. 2021), functional parietal cells in fundic-like organoids were transiently appeared and then lost during passaging, and mature chief cells were also absent in their system (Broda et al. 2019; McCracken et al. 2017). Furthermore, how multiple signaling pathways interact to regulate gastric epithelial cell differentiation is unclear. In this study, we successfully established organoid culture systems to achieve efficient differentiation of all gastric epithelial cells including pit cells, parietal cells, and chief cells. We found the key role of BMP signaling and its target Zbtb7b transcription factor in pit cell differentiation. Manipulation of BMP signaling and other signaling pathways can achieve distinct differentiation outcomes in gastric organoids. Our results provide an insight into the mechanisms underlying the regulation of gastric epithelial cell differentiation by niche signals in both human and mice and lay a foundation for future investigation and potential clinical use of gastric organoids.

## Results

### Establishment of gastric organoids for in vitro investigation of cell differentiation

Gastric glands in the corpus and antrum consist of various cell types, including pit cells, parietal cells, chief cells, enteroendocrine cells, and others, and these functional cells are differentiated from progenitor or stem cells (Barker et al. 2010; Han et al. 2019) **(**Fig. 1a**)**. To understand how the gastric epithelial cell differentiation is regulated, we cultured mouse gastric organoids in the complete medium (CM) containing EGF, BMP inhibitor (Noggin), R-spondin1, CHIR-99021, TGF-β inhibitor (SB431542), gastrin, FGF10 and ROCK inhibitor (Y-27632), as reported previously (Barker et al. 2010). In this system, we observed an enrichment of neck cells (GSII^+^) and proliferative cells (Mki67^+^), a limited number of pit cells (UEAI^+^) and chief cells (Gif^+^), but no parietal cells (HK-ATPase-β^+^, encoded by *Atp4b*) (Fig. 1b and Fig. S1a). Although parietal cells and chief cells were observed in the beginning of the gastric organoid culture, their numbers were decreased after passage (Fig. S1b-c). In contrast, a slight increase in proliferative cells was observed (Fig. S1c). Additionally, we also observed co-expression of Pcna and Mist1 in P0 gastric organoids (Fig. S1d), suggesting enhanced proliferative activity in Mist1^+^ chief cells under CM.

To better understand the cellular composition of gastric epithelial cells, we performed scRNA-seq on epithelial cells from mouse stomachs, including both the corpus and antrum (Fig. 1c). By clustering the cells based on previously reported lineage markers, we found that pit cells were enriched in the antrum, while chief and parietal cells were predominant in the corpus (Fig. 1d, Fig. S1e and Table S4−5). Compared to the antral and corpus tissues, the expression of differentiated cell markers was significantly decreased in the organoids, including pit cells, parietal cell, chief cells, and neck cells (Fig. S1f-g). Therefore, the present organoid culture system does not fully reflect the cellular composition in the tissue.

To optimize gastric organoid culture and elucidate the signaling mechanisms regulating gastric epithelial homeostasis, we investigated the signaling pathways underlying cell differentiation. By modulating pathways such as EGF, BMP4, and Wnt signaling through specific cytokines or small molecules, we have achieved the differentiation of pit cells, parietal cells, and chief cells in mouse gastric organoids. After two days in differentiation medium, a significant increase in the proportion of these cell types was observed (Fig. 1e-f). To analyze the cell composition in the organoids, we performed single-cell transcriptome analysis. A total of 8,747 cells from four organoid samples cultured under different conditions were analyzed, 10 distinct clusters of gastric epithelial cells were classified (Fig. 1g, Fig. S2a-b and Table S4−5). We observed a high proportion of proliferative cells and neck progenitors in the CM-cultured organoids, while pit cells were enriched in the pit cell differentiation medium (DM-Pit), more pit/parietal intermediate cells in the parietal cell differentiation medium (DM-Pa), and more chief cells and chief/neck intermediate cells in the chief cell differentiation medium (DM-C) (Fig. 1g and Fig. S2a-b). In addition, we observed a subgroup of cells expressing both the chief cell marker *Gif* and the neck cell marker *Muc6* (termed them as chief/neck intermediates), suggesting an intermediate state during cell differentiation (Fig. S2c). We also detected a group of cells co-expressing the pit cell markers *Muc5ac* and *Gkn1* and the parietal cell markers *Atp4b* and *Esrrg* (Fig. S2d-g), and termed them as pit/parietal intermediates. This finding aligns with a previous report suggesting that pre-pit cells may transition into pre-parietal cells (Karam 2010). By comparing the differentially expressed genes among the clusters, we identified a unique set of signature genes specific to pit-parietal intermediate cells, which included *Hsd17b2*, *Slc5a5*, *Igfbp5*, *Ces1f*, *Lypd8*, *Ly6d*, *Sptssb*, *Mal*, and *Crip1* (Fig. S2h). The pit/parietal intermediates may reflect the incomplete differentiation of parietal or pit cells in vitro or a transition from pre-pit to pre-parietal cells. To address whether these intermediates exist in vivo, we examined signature genes (Fig. S2h) in corpus glands using immunofluorescent staining under homeostasis and acute stomach damage conditions in mice. Only Crip1 exhibited strong staining, while other antibodies lacked specific signals. As shown in Fig. S3a, we induced acute tissue damage with a high dose of tamoxifen (0.2 mg/g mouse weight, single injection) and monitored the expression of Crip1, HK-ATPase-β (parietal cell marker), and UEAI (pit cell marker) during regeneration. Notably, Crip1 was detected on day 0, with several Crip1^+^cells co-expressing HK-ATPase-β and UEAI in the corpus gland under homeostasis (Fig. S3b). Interestingly, the number of Crip1^+^/HK-ATPase-β^+^ cells were increased at days 6 and 8 and then decreased after regeneration (Fig. S3c). Consistently, we confirmed the presence of *Atp4b* and *Crip1* co-expressing cells in parietal cells and pit cells in tissue (Fig. S3d). Importantly, cells co-expressing the parietal cell marker *Atp4b* and the pit cell marker *Muc5ac* were also observed in tissue (Fig. S3e). These findings suggest that pit/parietal intermediate cells may serve as progenitors for both pit and parietal cells in vivo and in vitro.

To compare cell similarities between organoids and tissues, we integrated the scRNA-seq data from mouse gastric epithelial tissues and 4 organoid samples and identified 12 gastric epithelial clusters in a total of 32,252 cells (Fig. S4a-c and Table S4−5). A high similarity of pit cells, neck cells and proliferative cells in the organoids and tissues was observed (Fig. S4d). The lineage markers for pit cells, proliferative cells, and neck cells exhibited comparable expression levels in both organoids and tissues, but parietal cell markers were apparently low in organoids (Fig. S4e). Moreover, most genes in pit cells, parietal cells, proliferative cells, and neck cells were overlapped between organoids and tissues (Fig. S4f and Table S6), indicating that our culture conditions effectively induce and maintain the characteristic of these cell types in vitro. Although HK-ATPase-β^+^ parietal cells were increased in organoids treated with DM-Pa (Fig. 1f), scRNA-seq revealed a lower expression of parietal cell markers, including *Atp4b*, *Atp4a*, and *Esrrg* (Fig. S4e), reflecting a reduced Pearson correlation score for parietal cells (Fig. S4d). Additionally, chief cells exhibited a lower correlation score (Fig. S4d), suggesting that additional factors are required for chief cell differentiation to reach the mature state.

To explore the effect of niche signals on epithelial cell differentiation, we analyzed signaling signature genes in the epithelial clusters (Table S7). The expression of BMP4 signature genes was notably high in parietal cells, pit cells, pit/parietal intermediates and tuft cells (Fig. S4g). Furthermore, WNT signature genes were enriched in proliferative cells, neck cells, neck progenitors, pit progenitors and chief/neck intermediates (Fig. S4h), consistent with the known function of neck cells and immature chief cells as reserve stem cells in gastric glands (Bockerstett et al. 2020; Stange et al. 2013). Additionally, EGF signature genes were highly expressed in pit cells, and TGF-β signature genes low in chief cells, immature chief cell and chief/neck intermediates (Fig. S4i-j).

### BMP4 induces differentiation of parietal cells and enteroendocrine cells

**Fig. 2:**
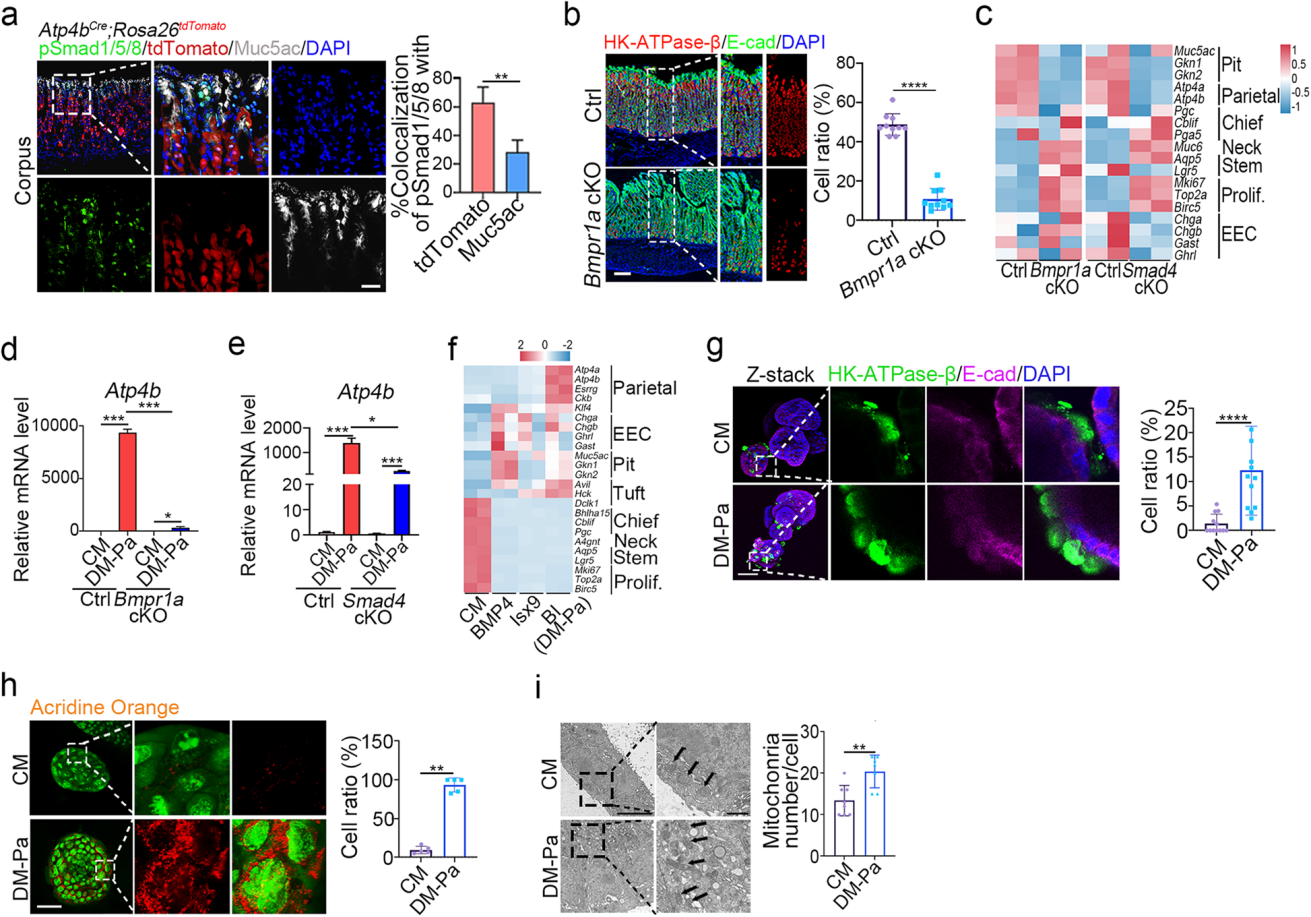
BMP4 promotes differentiation of parietal cells and enteroendocrine cells. **a** Immunofluorescent staining and quantification of corpus of *Atp4b*^*Cre*^*;Rosa 26 *^*tdTomato*^ mice for pSmad1/5/8, Muc5ac and E-cad (*n* = 3). Scale bars, 100 μm. **b** Immunofluorescent staining of HK-ATPase-β and E-cad, and quantification in mouse gastric glands in the corpus region of *Bmpr1a* cKO mice (*n* = 3). Scale bars, 100 μm. **c** Heatmap showing differential genes expression related to cell lineage markers in *Bmpr1a* or *Smad4* cKO mouse corpus glands, analyzed from bulk RNA-seq (*n* = 2). **d**-**e** The mRNA expression of *Atp4b* in mouse gastric organoids derived from control, *Bmpr1a* (**d**) or *Smad4* (**e**) cKO mice cultured under the indicated conditions for 2 days. **f** Heatmap showing differential expression of cell lineage markers in mouse gastric organoids cultured under the indicated conditions, analyzed from bulk RNA-seq (*n* = 2). **g** Immunofluorescent staining of HK-ATPase-β and E-cad, and quantification in mouse gastric organoids cultured under the indicated conditions (*n* = 11). Scale bars, 50 μm. Z-stack merged views were enlarged. **h** Acridine orange accumulation after histamine stimulation for 30 min in gastric organoids and quantification of acridine orange positive cells under the indicated conditions (*n* = 5). Scale bars, 50 μm. **i** Transmission electron microscopy and quantification of mitochondria numbers in mouse gastric organoids cultured under the indicated conditions for 2 days (*n* = 7). Enlarged images highlight organelle details within a cell, with arrows indicating mitochondria. Scale bars in left images, 5 μm; scale bars in right images, 1 μm. All immunofluorescence images were counter-stained with DAPI to show nuclei. Statistical significances were determined by unpaired multiple t test. *p < 0.05, **p < 0.01, ***p < 0.001, ****p < 0.0001

BMP4, secreted by mesenchymal cells surrounding the parietal cells in the isthmus region of mouse gastric glands, plays a crucial role in parietal cell differentiation (Bartfeld and Koo 2017; Kapalczynska et al. 2022). Withdrawal of Noggin, a BMP antagonist, led to a slight upregulation of parietal cell marker *Atp4b* in mouse gastric organoids (Fig. S5a). This observation was confirmed using the parietal cell-specific mice *Atp4b*^*Cre*^*;Rosa26*^*tdTomato*^, which showed the presence of pSmad1/5/8 signal within a substantial portion of tdTomato^+^ parietal cells and Muc5ac^+^ pit cells **(**Fig. 2a**)**. We blocked BMP signaling in gastric epithelial cells by *Cldn18*^*CreERT2*^-mediated conditional knockout (cKO) of the BMP receptor *Bmpr1a* or the downstream transducer *Smad4* (Fatehullah et al. 2021). In *Bmpr1a* or *Smad4* cKO mice, HK-ATPase-β-labeled parietal cells dramatically decreased, alongside a decline of parietal cell marker expression one month post tamoxifen-induced knockout (Fig. 2b-c and Fig. S5b). Using parietal cell differentiation medium (DM-Pa) to induce parietal cell differentiation also showed a decreased expression of parietal cell markers *Atp4b* and *Esrrg* in *Bmpr1a* or *Smad4* cKO mouse gastric organoids (Fig. 2d-e and Fig. S5c). In contrast, parietal cells exhibited lower expression of Wnt and EGF signatures (Fig. S4h-i). Withdrawal of EGF, Noggin, R-spondin1, and CHIR-9901 (-ENRC) led to a moderate increase in *Atp4b* expression, while BMP4 addition (-ENRC + B) significantly enhanced *Atp4b* expression (Fig. S5a). These data together support the role of BMP in promoting parietal cell differentiation.

The AMPK activator metformin has been shown to accelerate the maturation of parietal cells in mice (Miao et al. 2020). Although metformin increased *Atp4b* expression, it did not further enhance *Atp4b* expression in the present of BMP4 (BM) (Fig. S5d). Surprisingly, Isoxazole 9 (Isx-9), known for inducing differentiation of neuron cells and enteroendocrine cells in small intestinal organoids (Koh et al. 2015; Tsakmaki et al. 2020), improved parietal cell differentiation, and its combination with BMP4 (BI, DM-Pa) significantly upregulated parietal cell marker expression (Fig. 2f and Fig. S5d). Consistently, DM-Pa treatment also increased HK-ATPase-β^+^ parietal cells (Fig. 2g), and induced acidified cellular vesicles and mitochondria numbers (Fig. 2h-i), in agreement with the note that increased acidified cellular vesicles and mitochondria are found in functional parietal cells (McCracken et al. 2017; Miao et al. 2020). Isx-9 also activated the expression of enteroendocrine cell markers and expanded Chga^+^ and Gast^+^ enteroendocrine cells in gastric organoids (Fig. S5e-k). Although DM-Pa treatment reduced other cell lineages, such as Mki67^+^ proliferative cells, GSII^+^ neck cells and Aqp5^+^ antrum stem cells, it increased UEAI^+^ pit cells (Fig. S6). Taken together, BMP4 plus Isx-9 enhanced both parietal and enteroendocrine cell differentiation in mouse gastric organoids.

### Chief cell differentiation requires activation of Wnt signaling and suppression of TGF-β and BMP signaling

**Fig. 3:**
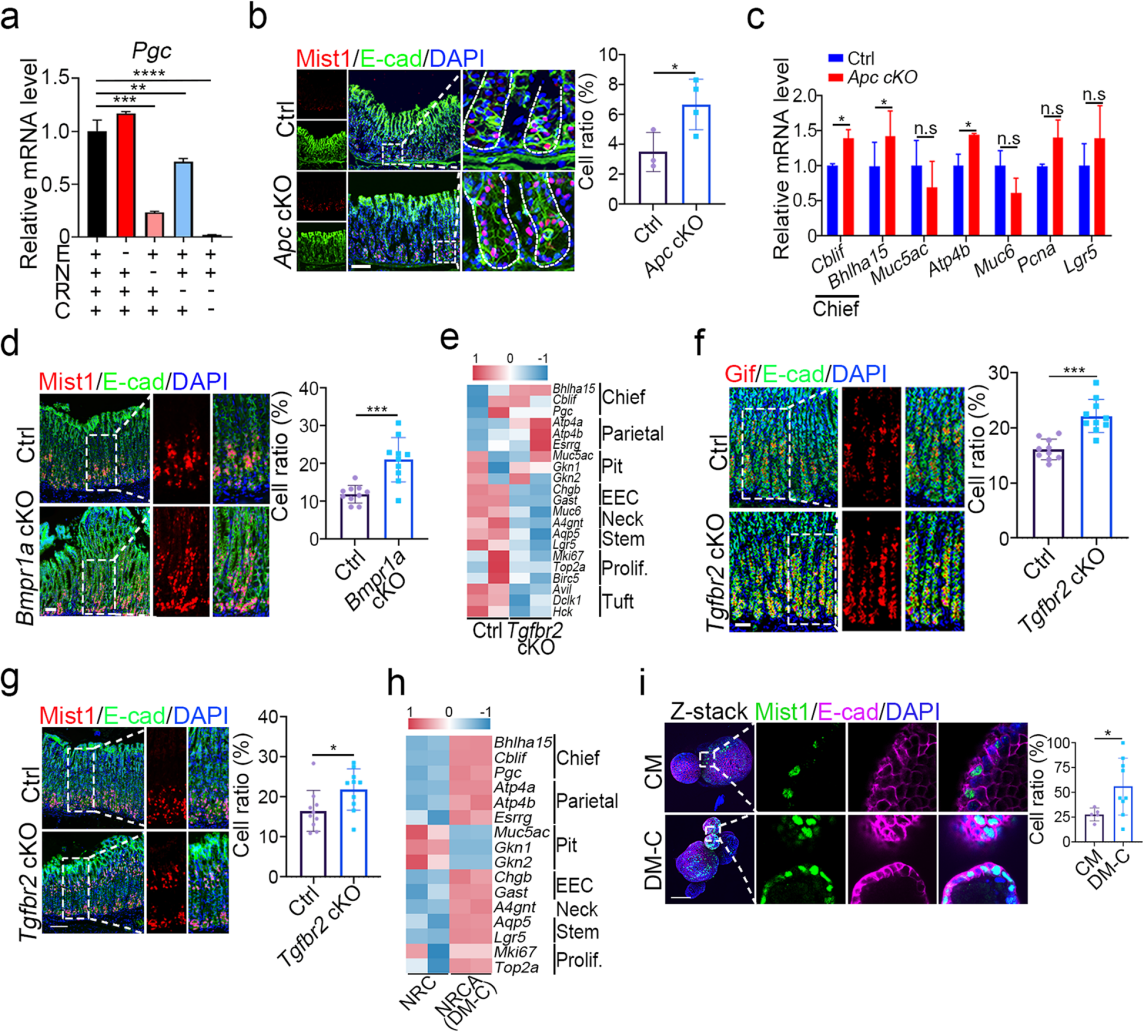
Chief cell differentiation requires activation of Wnt signaling and suppression of TGF-β and BMP signaling. **a** The mRNA expression of *Pgc* in mouse gastric organoids cultured under the indicated conditions for 2 days. E: EGF, N: Noggin, R: R-spondin1, C: CHIR-99021. **b** Immunofluorescent staining of Mist1 and E-cad, and quantification in mouse gastric glands in the corpus region of control or *Apc* cKO mice (*n* = 3). Scale bars, 100 μm. **c**, The mRNA expression of cell lineage markers in corpus glands of control or *Apc* cKO mice. **d** Immunofluorescent staining of Mist1 and E-cad, and quantification in mouse gastric glands in the corpus region of control or *Bmpr1a* cKO mice (*n* = 3). Scale bars, 100 μm. **e** Heatmap showing differential expression of cell lineage markers in corpus glands of control or *Tgfbr2* cKO mice, obtained from bulk RNA-seq (*n* = 2). **f**-**g**, Immunofluorescent staining of Gif, Mist1 and E-cad, and quantification in mouse gastric glands in the corpus region of control or *Tgfbr2* cKO mice (*n* = 3). Scale bars, 100 μm. **h** Heatmap showing differential expression of cell lineage markers in mouse gastric organoids cultured under the indicated conditions, obtained from bulk RNA-seq (*n* = 2). NRC: add Noggin, Rspondin1 and CHIR-99021 in basal culture medium; NRCA: addition of A83-01 in NRC medium. **i**, Immunofluorescent staining of Mist1 and E-cad, and quantification in mouse gastric organoids cultured under the indicated conditions (*n* = 7). Z-stack merged views with enlarged images. Scale bars, 50 μm. All immunofluorescence images were counter-stained with DAPI to show nuclei. Statistical significances were determined by unpaired multiple t test. *p < 0.05, **p < 0.01, ***p < 0.001, ****p < 0.0001

Chief cells located at the bottom of the gastric glands have a remarkable renewal time of over half a year and can serve as progenitor or reserve stem cells in the gastric epithelium (Douchi et al. 2021; Lee et al. 2022). The maintenance of chief cells is regulated by Wnt signaling, and both R-spondin1 and Wnt3a are concentrated in the bottom region of gastric glands (Sigal et al. 2017; Sigal et al. 2019). Withdrawal of R-spondin1 and CHIR-99021 and inhibition of Wnt signaling by IWP2 significantly reduced chief cell marker *Pgc* expression in mouse gastric organoids (Fig. 3a and Fig. S7a). Consistently, *Apc* cKO resulted in a significant increase of Mist1^+^ chief cells at the bottom of gastric glands, with minimal impact on Mki67-labeled proliferative cells in the isthmus region (Fig. 3b and Fig. S7b). The mRNA expression of chief cell markers, including *Cblif* and *Bhlha15* (Mist1), also increased in the stomachs of *Apc* cKO mice (Fig. 3c). However, *Pgc* expression remained unchanged following EGF withdrawal, suggesting that EGF signaling is dispensable for chief cell differentiation (Fig. 3a). Disruption of BMP signaling by *Bmpr1a* or *Smad4* cKO increased Mist1^+^ chief cells, GSII^+^ neck cells and Mki67^+^ proliferative cells (Fig. 3d and Fig. S7c-d). Accordingly, simultaneous treatment of R-spondin1, CHIR-99021 and Noggin effectively induced chief cell differentiation (Fig. S7a, e, f). Furthermore, consistent with the lower expression of TGF-β signature genes in chief cells of mouse stomach tissues (Fig. S7g), blockage of TGF-β signaling by *Tgfbr2* cKO resulted in an increased expression of chief cell markers including *Bhlha15*, *Cblif* and *Pgc* and more Gif^+^ and Mist1^+^ chief cells in gastric epithelial cells (Fig. 3e-g), but had no significant effect on the expression of neck cell markers and proliferative cell markers (Fig. S7h). Addition of the TGF-β inhibitor A83-01 to the chief cell differentiation medium (DM-C, NRCA) upregulated the expression of a part of chief cell marker genes in mouse gastric organoids, such as *Bhlha15*, *Cblif* and *Pgc* (Fig. 3h). DM-C also increased the proportion of Mist1^+^ chief cells and Aqp5^+^ antrum stem cells (Fig. 3i and Fig. S7i), but did not affect Mki67^+^ proliferative cells and GSII^+^ neck cells (Fig. S7j-k). Mature chief cells, indicated by zymogen granules (Bredemeyer et al. 2009), are rarely observed in these organoids (Fig. S7l), suggesting that additional factors are required for chief cells to reach maturity. Nonetheless, the above results together indicate that the differentiation of chief cells requires the activation of Wnt signaling and inhibition of TGF-β and BMP signaling.

### Pit cell differentiation is enhanced by BMP4 and EGF signaling

**Fig. 4:**
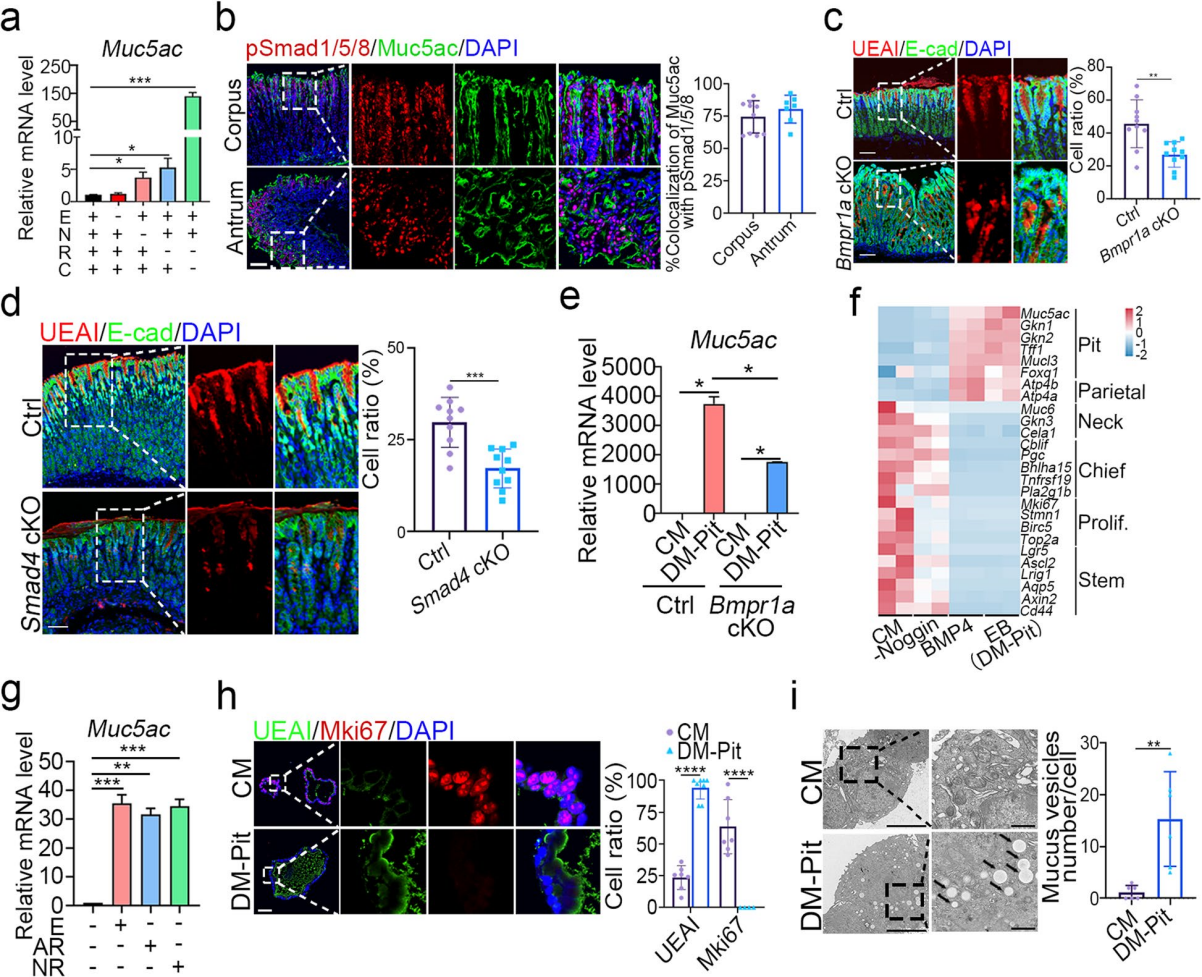
BMP4 and EGF signaling enhance Pit cell differentiation. **a** The mRNA expression of *Muc5ac* in mouse gastric organoids cultured under the indicated conditions for 2 day. E: EGF, N: Noggin, R: R-spondin1, C: CHIR-99021. **b** Immunofluorescent staining of pSmad1/5/8, Muc5ac and E-cad, and quantification in the corpus and antrum in wild type mice (*n* = 3). Scale bars, 100 μm. **c**-**d** Immunofluorescent staining of UEAI and E-cad, and quantification in the corpus region in control, *Bmpr1a* or *Smad4* cKO mice (*n* = 3). Scale bars, 100 μm. **e** The mRNA expression of *Muc5ac* in mouse gastric organoids cultured under the indicated conditions for 2 days. **f** Heatmap showing differential expression of cell lineage markers in mouse gastric organoids cultured under the indicated conditions, analyzed from bulk RNA-seq (*n* = 2). -Noggin: withdrawal of Noggin based on CM; BMP4, only add BMP4 based on the basal culture medium; EB, add EGF and BMP4 based on the basal culture medium. **g** The mRNA expression of *Muc5ac* in mouse gastric organoids cultured under the indicated conditions for 2 days. E: EGF, AR: AREG, NR: NRG1. **h** Immunofluorescent staining of UEAI, Mki67 and E-cad, and quantification in mouse gastric organoids cultured under the indicated conditions (*n* = 3). Scale bars, 50 μm. **i** Transmission electron microscopy and quantification of mucus vesicle numbers in mouse gastric organoids cultured under the indicated conditions for 2 days (*n* = 6). Enlarged images highlight organelle details within a cell, with arrows indicating mucus vesicles. Scale bars in left images, 5 μm; scale bars in right images, 1 μm. All immunofluorescence images were counter-stained with DAPI to show nuclei. Statistical significances were determined by unpaired multiple t test. *p < 0.05, **p < 0.01, ***p < 0.001, ****p < 0.0001

Wnt signaling has been shown to inhibit the differentiation of pit cells in human organoids culture system (Bartfeld et al. 2015), while BMP4 and EGF signaling have been reported to facilitate human pit cell differentiation (Wolffling et al. 2021). The removal of Noggin or R-spondin1 and the GSK3β inhibitor CHIR-99021 from CM resulted in a significant upregulation of the pit cell marker *Muc5ac* (Fig. 4a). This result is consistent with the scRNA-seq analysis of mouse gastric epithelial cells revealing that Wnt signature genes showed lower expression in pit cells but higher expression in proliferative cells and neck cells (Fig. S4h), suggesting that Wnt signaling is not necessary for pit cell differentiation. Secondly, the expression of BMP4 signature genes was high in pit cells and parietal cells (Fig. S4g), which is supported by immunostaining showing co-localization of pSmad1/5/8 with Muc5ac in the upper region of mouse gastric glands (Fig. 4b). Inhibition of BMP signaling led to a decreased expression of pit cell markers, including *Muc5ac*, *Gkn1*, and *Gkn2* (Fig. 2c), which was consistent with the data showing that UEAI-labeled pit cells were significantly decreased in the stomachs of *Bmpr1a* KO and *Smad4* KO mice (Fig. 4c-d). In the pit cell differentiation medium (DM-Pit), *Muc5ac* expression was also reduced in *Bmpr1a* cKO gastric organoids (Fig. 4e). Conversely, BMP4 significantly increased the expression of pit cell markers such as *Muc5ac*, *Gkn1*, *Gkn2*, *Tff1*, and *Mucl3* in mouse gastric organoids (Fig. 4f). The addition of EGF enhanced the effect of BMP4 on pit cell differentiation (Fig. 4f). Other EGF family members, including AREG (AR) and NRG1 (NR), exhibited similar effects on pit cell differentiation as EGF (Fig. 4g). The population of UEAI^+^ pit cells significantly increased (~ 90%) in gastric organoids cultured in the DM-Pit medium, accompanied by decreased expression of other lineage markers such as Mki67 (proliferative cells), GSII (neck cells) and Aqp5 (antrum stem cells) (Fig. 4h and Fig. S8). Additionally, the number of mucus vesicles in pit cells increased in organoids cultured in the DM-Pit medium (Fig. 4i). These data together suggest that simultaneous activation of EGF and BMP4 signaling is critical for the differentiation of functional pit cells.

### Zbtb7b is critical for pit cell differentiation

**Fig. 5:**
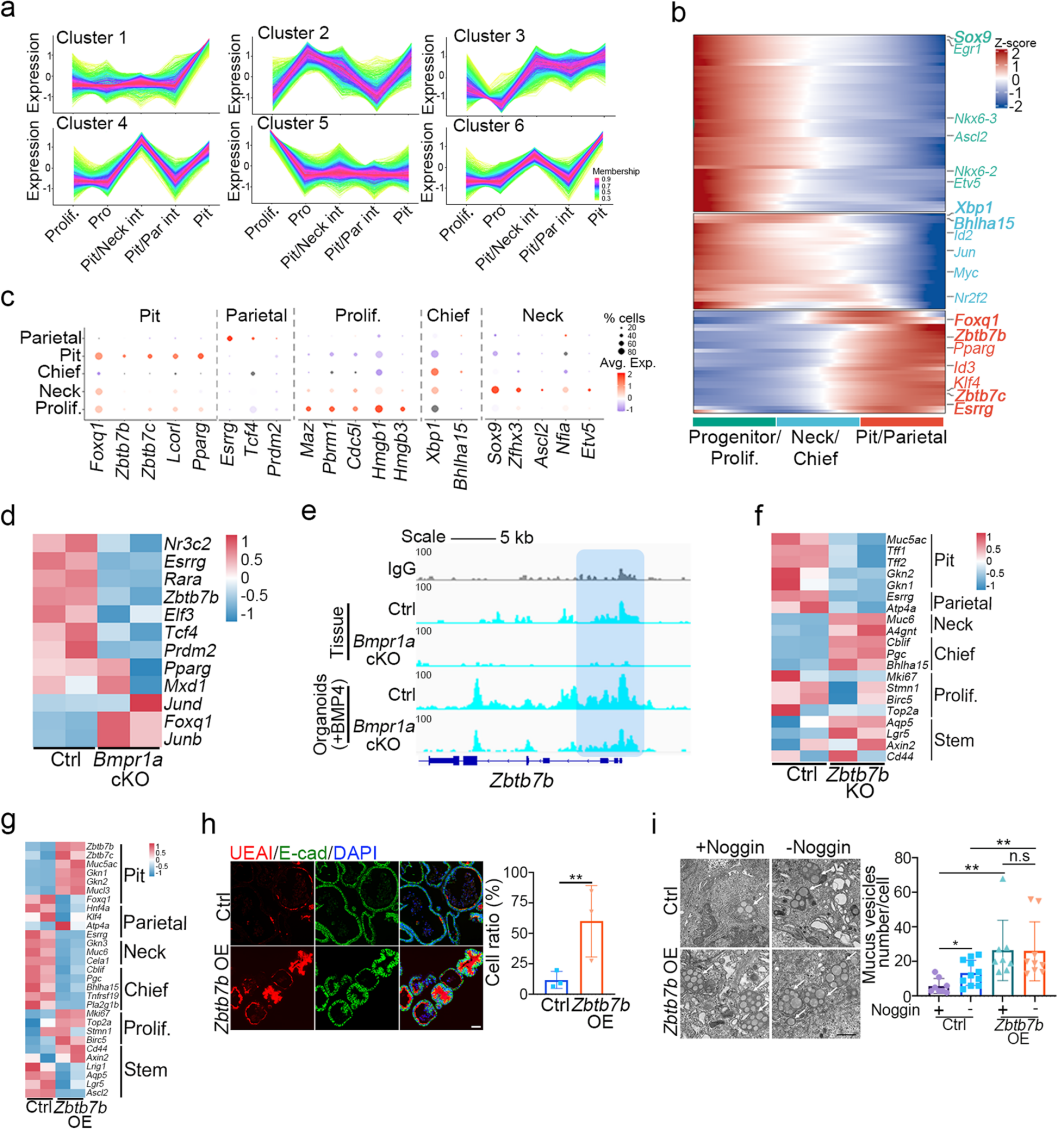
Zbtb7b is critical for pit cell differentiation. **a** Gene expression trends during pit cell differentiation were derived from scRNA-seq data in mouse gastric organoids (Fig. 1g). Clusters 1 and 6 represent upregulated genes during pit cell differentiation. Prolif.: proliferative cell, Pro: progenitor, Pit/Neck int: pit/neck intermediates, Pit/Parietal int: pit/parietal intermediates, Pit: pit cell. **b** Pseudotime analysis derived from mouse tissue scRNA-seq data (Fig. 1d) revealed transcription factors enriched in different cell types. The X axis represents cell types, and the Y axis displays specifically expressed transcription factors in these cell types. **c** Dot plot of screened transcriptional factors related to different types of cells. **d** Heatmap showing expression of 12 candidate genes in the corpus of control and *Bmpr1a* cKO mice. **e** Cut&Tag analysis displaying Smad1 binding peaks on the Zbtb7b promoter in control and *Bmpr1a* cKO gastric tissue or organoids treated with BMP4. **f**-**g** Heatmap showing differential expression of cell lineage markers in control, *Zbtb7b* KO or overexpressing mouse gastric organoids cultured for 2 days (*n* = 2). **h** Immunofluorescent staining of UEAI and E-cad, and quantification in control or Zbtb7b OE mouse gastric organoids (*n* = 3). Scale bars, 50 μm. **i** Transmission electron microscopy images and quantification of mucus vesicle numbers in control or Zbtb7b OE mouse gastric organoids, cultured for 2 days under specified conditions (*n* = 4). Noggin: 50 ng/ml. Arrows indicating mucus vesicles. Scale bar, 1 μm. All immunofluorescence images were counter-stained with DAPI to show nuclei. Statistical significance was determined by unpaired multiple t-test. *p < 0.05, **p < 0.01

The differentiation of pit cells is enhanced by BMP signaling (Fig. 4f, h, i), but the underlying mechanism remains unclear. We scrutinized the scRNA-seq data from mouse organoids to delineate gene expression patterns associated with pit cell differentiation and found that several genes in clusters 1 and 6 exhibit an upregulated expression trend in concert with the differentiation process of pit cells (Fig. 5a). Additionally, based on the reported transcription factors that function in gastric tissues, we identified lineage-specific transcription factors by analyzing the scRNA-seq data from mouse stomach tissues (Fig. 5b-c). For instance, the well-characterized parietal cell-associated transcription factor *Esrrg* (Adkins-Threats et al. 2024), and chief cell-related *Xbp1* and *Bhlha15* (Bredemeyer et al. 2009), were identified in our analysis. Subsequently, we focused on transcription factors specific to pit cell differentiation. Integrating upregulated genes during pit cell differentiation in organoids with tissue-specific transcription factors, we pinpointed 12 transcription factors, including *Zbtb7b*, *Pparg*, and *Foxq1*, which are potentially involved in pit cell differentiation (Fig. S9a). Aligning with our finding that BMP signaling promotes pit cell differentiation, 10 of these genes showed a consistent decrease in expression in the stomachs of *Bmpr1a* cKO mice (Fig. 5d). Furthermore, Cut&Tag analysis revealed that Smad1 bound to the Zbtb7b promoter, with reduced binding observed in *Bmpr1a* cKO gastric epithelial cells and organoids (Fig. 5e). ChIP-qPCR confirmed the binding of Smad1 to the *Zbtb7b* promoter, and luciferase reporter assay demonstrated the Zbtb7b promoter responded to BMP4 treatment (Fig. S9b-c). Immunofluorescence analysis unveiled that Zbtb7b was mainly enriched in the top region of gastric glands in the corpus and a large part of the gastric gland in the antrum, co-localized with most UEAI-labeled pit cells (Fig. S9d). Additionally, Zbtb7b^+^ cells were diminished in *Bmpr1a* cKO stomach, and the induction of Zbtb7b expression by BMP was disrupted in *Bmpr1a* KO gastric organoids (Fig. S9e-f). These data strongly suggest that *Zbtb7b* is a BMP target that specifically expressed in pit cells.

To explore the role of *Zbtb7b* in pit cell differentiation, we conducted *Zbtb7b* KO in gastric organoids and assessed the expression of cell lineage markers (Fig. 5f and Fig. S10a). *Zbtb7b* KO significantly decreased the expression of the pit cell markers *Muc5ac* and *Gkn1*, while increasing the neck cell marker *Muc6* and the stem cell markers *Aqp5* and *Lgr5* (Fig. 5f). Moreover, BMP4-induced expression of *Muc5ac* was inhibited in *Zbtb7b* KO organoids (Fig. S10b). The number of mucous vesicles also decreased after Zbtb7b knockout (Fig. S10c). Conversely, overexpression of Zbtb7b in gastric organoids increased the expression of *Muc5ac* and *Gkn1* as well as UEAI^+^ or Muc5ac^+^ pit cells (Fig. 5g-h and Fig. S10d-f). Mucous vesicles were significantly enriched in gastric organoids with Zbtb7b overexpression, even when treated with the BMP inhibitor Noggin (Fig. 5i). Consistently, Cut&Tag analysis of Zbtb7b bound to the promoter regions of *Muc5ac* and *Gkn1*, which was reduced in *Bmpr1a* KO cells (Fig. S10g). These data together indicate that the zinc finger transcription factor Zbtb7b mediates BMP signaling to induce pit cell differentiation.

### Differentiation of human gastric epithelial cells

To investigate the differentiation of human gastric epithelial cells, we first characterized the cell composition in the human stomach by scRNA-seq analysis of seven datasets derived from 3 corpus and 4 antrum samples (Fig. S11a). As shown in Fig. S11b, all the gastric epithelial cell types were detected in the human stomach (Fig. S11b-d and Table S4−5). Using the same culture condition (CM) as for mouse gastric organoids, we successfully generated human gastric organoids. Similar to mouse organoids, human organoids contained a high amount of MKI67^+^ proliferative cells and MUC6^+^ neck cells, but pit cells (UEAI^+^) and parietal cells (HK-ATPase-β^+^) was limited (Fig. S11e). The expression levels of differentiated cell markers were also markedly reduced in the organoids compared to the corresponding tissue samples (Fig. S11f-g).

**Fig. 6:**
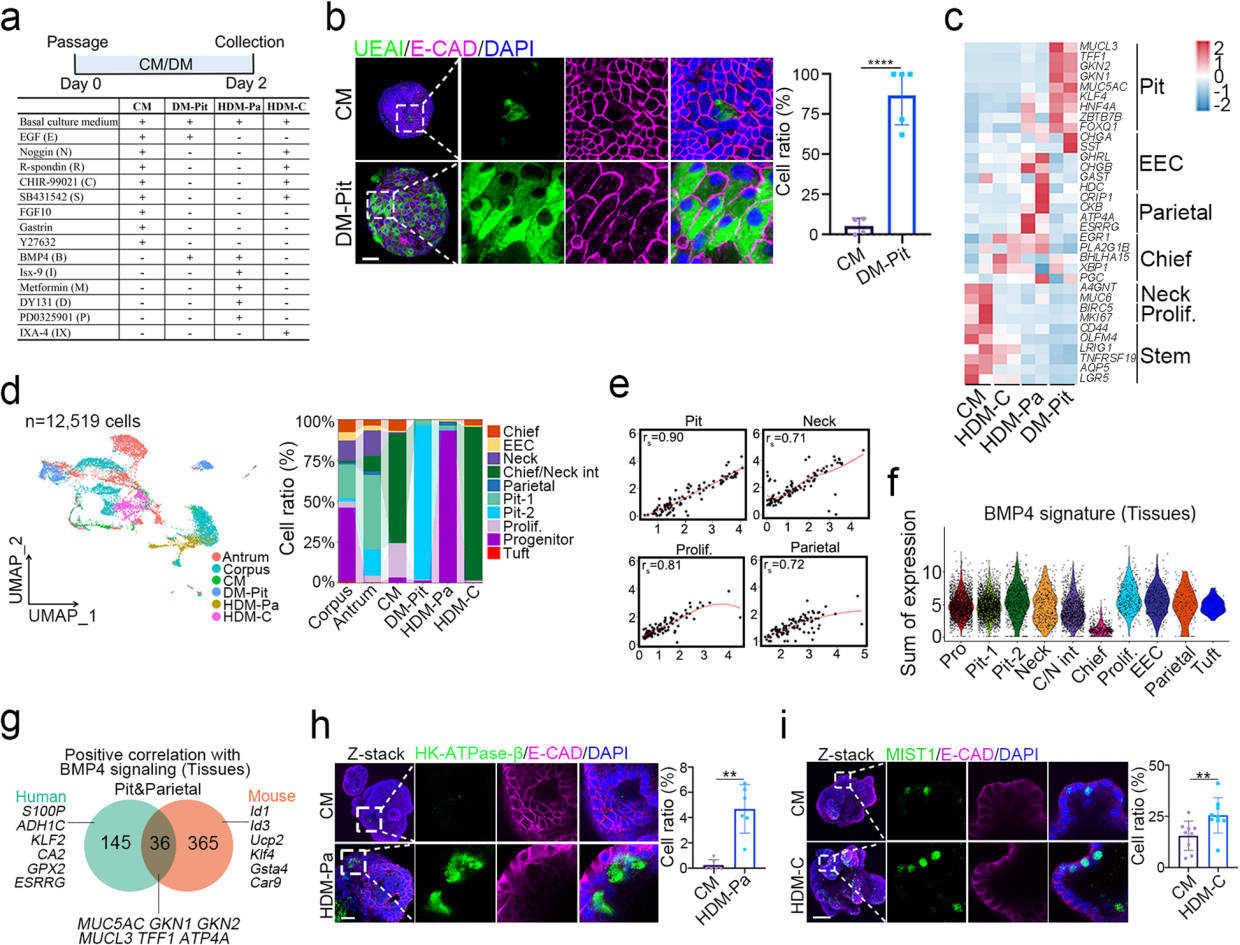
Differentiation of human gastric epithelial cells. **a** A schematic illustration of human gastric organoids cultured under different media for 2 days, along with a comparison of medium composition in the complete medium (CM) and cell differentiation media for pit cell (DM-Pit), parietal cell (HDM-Pa) and chief cell (HDM-C) in human gastric organoids. The detail component for basal culture medium and the concentrations of these growth factors or molecular include in Table S1. **b** Immunofluorescent staining of UEAI and E-CAD, and quantification in human gastric organoids cultured under the indicated conditions (*n* = 4). Scale bars, 50 μm. **c** Heatmap showing differential expression of cell lineage markers in human gastric organoids cultured under the indicated conditions, analyzed from bulk RNA-seq (*n* = 2). HDM-C: chief cell differentiation medium; HDM-Pa: parietal cell differentiation medium. **d** UMAP visualization of integrated human gastric organoids cultured under the indicated conditions (*n* = 4), human corpus (*n* = 3) and human antrum (*n* = 4), along with corresponding cell ratio (right panel). Prolif.: proliferative cells, Chief/Neck int: Chief/Neck intermediates. **e** The Spearman correlation analysis of scRNA-seq expression (UMI count) of characteristic genes for pit cells, neck cells, parietal cells and proliferative cells in tissues (X axis) and organoids (Y axis). **f** Violin plot showing the expression of BMP4 signature in different clusters of epithelial cells of human stomach tissues. **g** Venn diagrams displaying genes in pit and parietal cell lineages positively correlated with BMP4 signaling in human stomach tissues. **h**-**i** Immunofluorescent staining of HK-ATPase-β, MIST1 and E-CAD, and quantification in human gastric organoids cultured under the indicated conditions (*n* = 6). Scale bars, 50 μm. The left images represent z-stack merged views with enlarged images. All immunofluorescence images were counter-stained with DAPI to show nuclei. Statistical significance was determined by unpaired multiple t-test. *p < 0.05, **p < 0.01, ****p < 0.0001

To recapture cell composition in gastric tissue, we manipulated the organoid culture conditions by changing various growth factors or small molecules (Fig. 6a). Similar to mouse organoids, the addition of EGF and BMP4 (DM-Pit) to human gastric organoids resulted in increased expression of pit cell markers such as *MUC5AC* and *GKN2* (Fig. S11h), increased UEAI^+^ pit cells (Fig. 6b), and reduced MKI67^+^ proliferative cells (Fig. S11i). We also implemented distinct conditions to achieve differentiation of parietal cells (BIMDP: BMP4, Isx-9, Metformin, DY131, and PD0325901, refer to HDM-Pa) and chief cells (NRCSI: Noggin, R-spondin1, CHIR-99021, SB431542, and IXA4, refer to HDM-C) in human gastric organoids, and achieved an enrichment of corresponding marker genes in these conditions (Fig. 6c).

To further investigate the molecular mechanism underlying cell differentiation in human gastric epithelium, we conducted scRNA-seq analysis of human gastric organoids and integrated the data with the one generated from human gastric epithelial tissues (Fig. S12a). Using established markers, we identified 10 distinct cell clusters (Fig. 6d and Fig. S12b). The proportion of pit-2 cells were increased in the DM-Pit medium, parietal cells and progenitors in HDM-Pa, and chief/neck intermediates in HDM-C, compared to those in CM (Fig. 6d). Notably, organoids cultured with DM-Pit displayed different pit cell clusters (pit-1 and pit-2) with varying levels of *MUC5AC* expression, suggesting that these cells are in different stages of maturation (Fig. S11 d and Fig. S12c). Due to the limited number of parietal cells captured and the sequencing depth of scRNA-seq, a low expression of the parietal cell marker *ESRRG* was observed in the C9 parietal cell cluster and C1 progenitor (Fig. S12 d). Additionally, the C4 cluster co-expressed the chief cell marker *PGC* and neck cell marker *MUC6*, perhaps being chief/neck intermediates (Fig. S12e). The correlation in pit, parietal, neck, and proliferative cells between the organoids and human tissues was generally high (Fig. 6e), except for chief cells, which exhibited a lower correlation (Fig. S12f), indicating that cell differentiation is recaptured in the organoid systems.

The signaling pathways controlling gastric epithelial cell differentiation have both conserved and distinct aspects between humans and mice. Both human and mouse gastric epithelial cells exhibited enrichment of BMP signature genes in pit and parietal cells (Fig. S4g and Fig. 6f), indicating a conserved role for BMP signaling in these lineages. Although some BMP target genes were shared between species, many were distinct (Fig. 6g and Table S11). Among the 36 shared genes, there was a notable enrichment for pit cell markers such as *MUC5AC*, *GKN1*, *GKN2*, and *TFF1*, reflecting similarities in pit cell differentiation between the two species. However, 145 genes were specifically expressed in human pit and parietal cells, while 365 genes were specific to mouse pit or parietal cells. Similar to mouse gastric epithelial cells, human gastric epithelial cells also exhibited a high expression of Wnt signature genes in proliferative cells, followed by neck lineage (Fig. S13a). Moreover, among the shared 71 genes, including *MKI67*, *TOP2A* and *BIRC5*, exhibited a significantly positive correlation to Wnt signaling (Fig. S13b). Except in chief cells, the EGF signature genes were observed in all other cell types of human gastric epithelial cells (Fig. S13c). Among them, 7 genes were conserved in human and mouse pit cells (Fig. S13d), all of which could be new factors involved in EGF signaling to modulate pit cell differentiation. In both human and mouse gastric epithelial cells, we observed that chief cells displayed the lowest expression of TGF-β signature genes (Fig. S7g and Fig. S13e). These data together indicate that TGF-β signaling may inhibit chief cell differentiation.

### Distinct signaling controls differentiation of parietal cells and chief cells in human and mouse

Although the composition of gastric epithelial cells is similar between human and mouse, the differentiation of some cell types may require different factors. EGF and BMP4 induced pit cell differentiation in both human and mouse gastric organoids (Fig. 4f, Fig. 6b-c and Fig. S11h). Although the BI medium (BMP and Isx-9) significantly promoted parietal cell differentiation in mouse gastric organoids, it was less effective in inducing human parietal cell differentiation (Fig. 2d-i, Fig. S5c-d and Fig. S14a). The activation of estrogen-related receptor gamma (ESRRG), important for parietal cell marker expression, along with the inhibition of EGF signaling, showed benefits for parietal cell differentiation (Alaynick et al. 2010; McCracken et al. 2017). We also examined the effects of metformin, DY131 (ESRRG activator), and PD0325901 (MEK inhibitor) on human gastric organoids to evaluate their impact on parietal cell differentiation (Fig. S14a). Treatment with each molecule individually resulted in increased *ATP4B* expression, and combination of these small molecules with BI slightly further enhanced *ATP4B* expression (Fig. S14a). However, immunostaining showed that organoids treated with HDM-Pa (contains BMP4, Isx-9, metformin, DY131 and PD0325901) apparently expanded the number of HK-ATPase-β^+^ cells (Fig. 6h), as well as increased the expression of EZRIN, another parietal cell marker (Fig. S14b). Notably, HDM-Pa decreased MKI67^+^ proliferative cells (Fig. S14c).

Although the differentiation of chief cells in mouse gastric epithelial cells depended the activation of Wnt signaling (Fig. 3a-c), Wnt3a even reduced the expression of the chief cell marker *MIST1* in human gastric organoids (Fig. S14d). Considering that *XBP1* is a gene involved in chief cell differentiation (Huh et al. 2010), we supplemented the mouse chief cell differentiation medium (DM-C) with IXA4, an agonist of XBP1, into human gastric organoids (Fig. 6a), and observed that this treatment led to a significant upregulation of *MIST1* (*BHLH15*) (Fig. S14d). Consistently, the number of MIST1^+^ chief cells significantly increased in human organoids cultured in the NRCSI medium (HDM-C) (Fig. 6i), while MUC6^+^ neck cells remained unchanged (Fig. S14e). These data suggest that more complex regulatory network is required for differentiation of human parietal cells and chief cells.

## Discussion

**Fig. 7:**
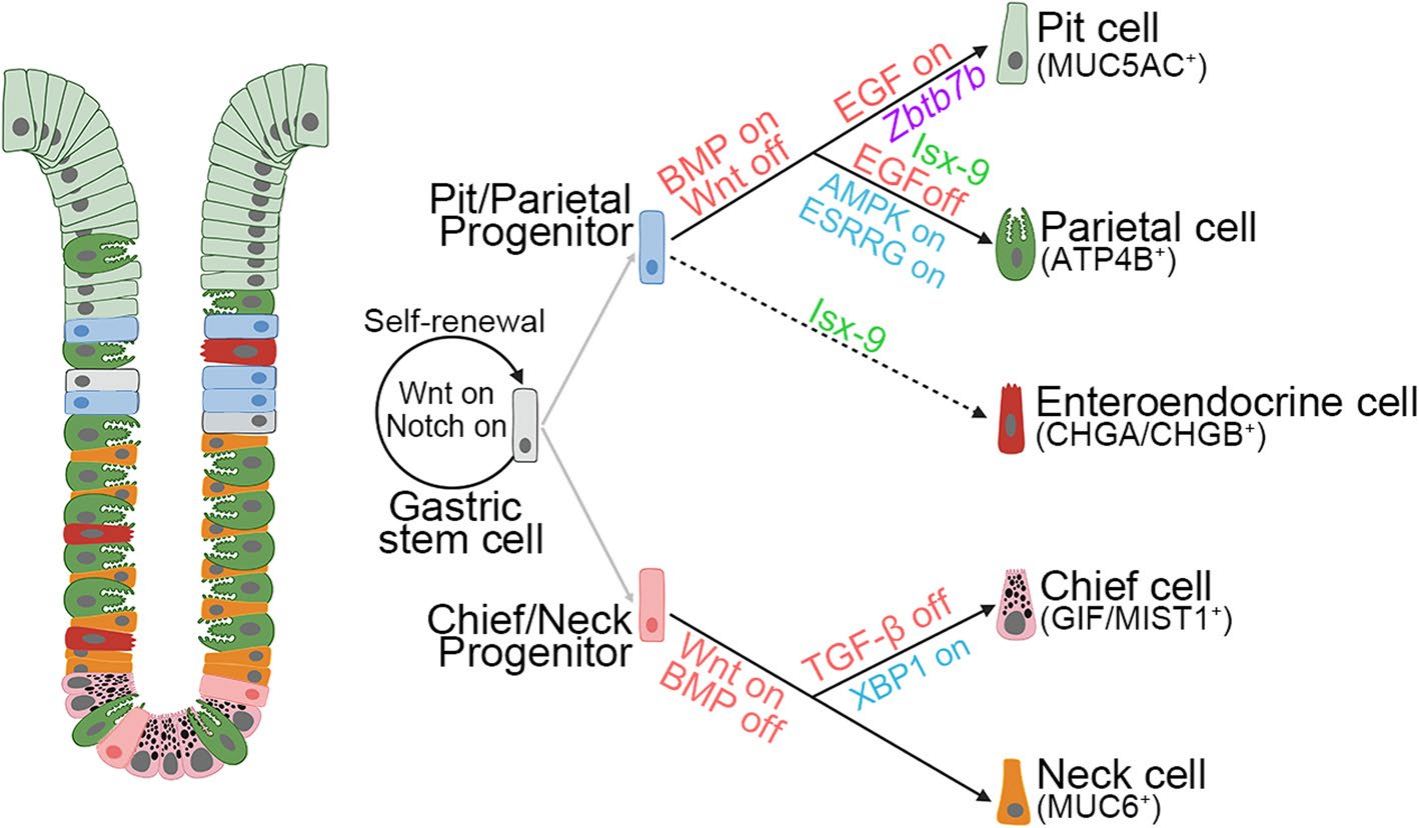
Schematic illustration of the differentiation paths of gastric epithelial cells and signaling regulation. Manipulation of specific signaling pathways is indicated by light red text. The transcriptional factor Zbtb7b controls pit cell differentiation. The small molecule Isx-9 triggers the differentiation of parietal and enteroendocrine cells. Notably, in human gastric organoids, specific activation of AMPK, ESRRG, and XBP1 is required for various lineage specification and highlighted in blue text

Although the cellular composition of the gastric epithelium is well known, how generation of these constituent cells is regulated by the niche factors is largely unclear. In this study, we systematically dissected the roles of the niche-derived factors in generation of the mature cells and found that BMP, EGF, Wnt and TGF-β play critical roles in the cell lineage specification (Fig. 7). For instance, BMP on, EGF on, and Wnt off lead to pit cell differentiation, with the novel regulator Zbtb7b controlling this process by mediating BMP signaling. BMP on, combined with the small molecule Isx-9, promotes the differentiation of progenitors into both parietal and enteroendocrine cells in mouse gastric organoids. BMP off, TGF-β off, and Wnt on promote chief cell differentiation. Furthermore, additional signaling pathways are required for differentiation of human parietal and chief cells.

Stomach organoids can be generated from adult gastric glands or pluripotent stem cells in both human and mouse. Although the composition of the culture medium vary in different studies, the key growth factors and molecules, such as EGF, Noggin, R-spondin1, Wnt activators (Wnt3a or CHIR-99021), FGF10, and gastrin, are used in gastric organoid cultures of human and mice (Seidlitz et al. 2021). The complex in vivo environment includes various cell types, such as fibroblasts, immune cells, and endothelial cells, all of which could influence the differentiation of gastric epithelial cells via different ways including secreting growth factors. In contrast, externally added growth factors and small molecules in cultured organoids are hard to fully mimic the in vivo microenvironment and may result in a more rapid cell differentiation at intermediate stages. This is consistent with our observation that a high proportion of proliferative cells were found in the complete medium.

In line with previous studies (Schumacher et al. 2015), we observed that the proportion of pit cells was low in the expansion media, EGF and BMP substantially increased pit cells in mouse gastric organoids (from 20 to 90%). Furthermore, we found that activation of EGF and BMP signaling is also required for human pit cell differentiation. Although activation of BMP signaling has been reported to enhance pit cell differentiation (Wolffling et al. 2021), the specific transcriptional mechanisms remain unclear. Foxq1 has been shown to be a key factor essential for the expression of *Muc5ac*, but other pit cell markers including *Gkn1*, *Gkn2*, and *Tff1* showed no change after *Foxq1* knockout in mice (Verzi et al. 2008). In addition, *Foxq1* expression was increased following *Bmpr1a* cKO in the mouse stomach, in contrast to the decreased expression of pit cell markers such as *Muc5ac*, *Gkn1*, and *Gkn2*, indicating that Foxq1 is unlikely to mediate BMP-dependent pit cell differentiation. Based on scRNA-seq screening and functional validation assays, we identified Zbtb7b as a key regulator of pit cell differentiation. Zbtb7b, also known as THPOK (T-helper-inducing POZ/Kruppel-like factor), plays a crucial role in CD4^+^ and CD8^+^ T-cell lineage determination in the thymus (Cheng et al. 2021). Previous studies have linked low ZBTB7B expression with a high risk and increased immune cell infiltration in gastric cancer patients (Cui et al. 2022). Although Zbtb7a/b have been identified as BMP downstream targets in epiblast stem cells (Yu et al. 2020), our study is the first to link Zbtb7b specifically to pit cell differentiation in the stomach, providing a novel mechanistic insight into how BMP signaling governs gastric epithelial differentiation.

Using organoids from adult gastric tissue, our study uncovers variations in responsiveness to distinct signaling during parietal cell differentiation in human and mouse. It is difficult to maintain parietal cells in vitro as they are gradually lost during passaging of Matrigel-based 3D culture and human pluripotent stem cell-derived organoids (Bartfeld et al. 2015; McCracken et al. 2017; Schumacher et al. 2015). Metformin has been shown to enhance the differentiation of parietal cells by activating the AMPK/PGC-1α pathway and improving mitochondrial maturation in mice with a limited effect (Miao et al. 2020). We found that treatment of BMP4 and Isx-9 greatly induced parietal cell differentiation in mouse gastric organoids. Isx-9 has been reported to stimulate intestinal enteroendocrine cell and neural differentiation (Tsakmaki et al. 2020). How Isx-9 and BMP4 work together to regulate parietal cell differentiation remains unclear, but it is possible that some genes vital for neural differentiation may be also required for parietal cell differentiation. In contrast to mouse gastric organoids, activation of ESRRG and AMPK is additionally required for the expression of the parietal cell marker *ATP4B* in human gastric organoids.

Chief cells, originally recognized as reserve stem cells, play a role in the regeneration of the mouse stomach (Lee et al. 2022; Stange et al. 2013). These cells also undergo de-differentiation into neck cells and subsequently initiate abnormal proliferation in human gastritis (Li et al. 2021; Meyer and Goldenring 2018). However, the mechanism governing chief cell differentiation by signaling pathways interaction is poorly understood. Our data revealed the inhibitory effect of TGF-β signaling on chief cell differentiation. Furthermore, our data unveiled that the balance of Wnt, BMP and TGF-β signaling is crucial in maintaining the characteristics of chief cells: Wnt signaling is vital for their maintenance, while BMP4 and TGF-β hinder their differentiation. It has been suggested that chief cells can be derived from neck cells in mice, and their maturation requires the expression of key transcription factors such as *Xbp1* and *Mist1 *(Bredemeyer et al. 2009; Huh et al. 2010; Lennerz et al. 2010). Consistent with this, we observed that the activation of XBP1 by IXA4 increased the expression of *MIST1* in human gastric organoids. However, chief cells in the organoids cultured in the differentiation media exhibited an immature status in both human and mouse with rare presence of pepsinogen granules. Therefore, other factors that are required to achieve chief cell maturation in vitro await further elucidation.

While mice possess a forestomach with stratified squamous epithelium that is absent in human, the corpus and antral regions exhibit analogous anatomy, similar cell composition and function in both species (Kim and Shivdasani 2016; Willet and Mills 2016). However, gene expression shows species variation, especially for parietal, chief, and neck cells (Busslinger et al. 2021). Examples include *GIF*, *CCKBR*, and *CPA2* predominantly in human parietal cells, while *Apoa1* and *Ppp1r1b* are mouse parietal cells. Mouse chief cells express *Clps*, *Gif,* and *Pla2 g1b*, while *LIPF* and *PGA5* are human-specific. Our study uncovers that BMP signaling enhances pit and parietal cell differentiation, EGF signaling aids pit cell differentiation, and Wnt signaling promotes chief and neck cell differentiation in both species. Additionally, the antral stem cell marker *AQP5* and key transcriptional factor of chief cells *XBP1* exhibit a stronger correlation with Wnt signaling in human compared to mouse. The pit cell marker *Foxq1* correlates positively with EGF signaling in mouse, whereas *MUC5AC*, *MUCL3* and *TFF1* show a correlation with EGF signaling in human. Dissection of the detailed signaling pathways and molecules that regulate cell lineage differentiation would greatly benefit the treatment of related gastric diseases.

## Materials and methods

### Human stomach tissue collection and ethical statement

The human antrum tissue, located approximately 2~3 cm adjacent to the pylorus, and corpus tissue taken from the central part of the corpus, were collected from patients undergoing sleeve gastrectomy at the Department of General Surgery, The First Affiliated Hospital of Nanjing Medical University, Nanjing, China. The study was conducted with prior approval from the Ethics Committee of The First Affiliated Hospital of Nanjing Medical University, with the approval number 2017-SR-171.A2. The samples were provided in an anonymized form to protect patient privacy. A total of 6 patients with a body mass index (BMI) greater than 31 kg/m^2^ were included in this study (Table S2).

### Animals

The *Cldn18*^*CreERT2*^ mice were obtained from GemPharmatech; the *Atp4b*^*Cre*^ mice, *Smad4*^*fl/fl*^, *Tgfbr2*^*fl/fl*^ mice were provided by Prof. Xiao Yang; *Bmpr1a*^*fl/fl*^ mice were provide by Yuji Mishina (Mishina et al. 2002); *Rosa26*^*loxp−stop−loxp−Cas9−EGFP*^ mice from Dr. Jianwei Wang; *Apc*^*fl/fl*^ and *Rosa26*^*tdTomato*^ mice were obtained from the Jackson Laboratory. All mice were housed and maintained at the Tsinghua University Animal Facility. The experimental procedures were conducted in accordance with the guidelines and protocols approved by the Institutional Animal Care and Use Committee (IACUC) of Tsinghua University, with the approval number 19-YGC. For Cre induction, mice received intraperitoneal injections of tamoxifen dissolved in oil at 20 mg/ml, 100 μl per injection, for four consecutive days. Control mice (floxed mice not crossed with Cldn18-CreERT2) were administered the same tamoxifen dosage. Analysis was performed one-month post-induction. Intraperitoneal injections of tamoxifen (40 mg/ml in oil, 0.2 mg/g mouse weight) were given to 8~10-week-old wild-type mice to induce acute stomach damage. The analysis was performed on days 0, 6, 8, and 14 post-injection.

### Antibodies

The following antibodies were used in this study: rabbit anti-Aqp5 (1:200, ab92320; Abcam), rabbit anti-Ki67 (1:200, ab15580; Abcam), rabbit anti-Muc5ac (1:300, ab3649; Abcam), rabbit anti-chromogranin A (1:300, ab15160; Abcam),rabbit anti-MUC6 (1:200, ab223846; Abcam), mouse anti-DYKDDDDK Tag (1:1000, 8146S; Cell Signaling), mouse anti-GAPDH (1:2000, TA-08; ZSGB-BIO), mouse anti-β acting (1:2000, TA-09; ZSGB-BIO), rabbit anti-E-cadherin (1: 300, 3195; Cell Signaling), rabbit anti-pSmad1/5/8 (1:200, 13820 s; Cell Signaling), rabbit anti-MIST1 (1:200, 14896S; Cell Signaling), mouse anti-HK-ATPase-β (1:300, sc-374094; Santa Cruz), mouse anti-Gif (1: 300, sc-514523; Santa Cruz), mouse anti-PCNA (1: 300, sc-56; Santa Cruz),rabbit anti-gastrin (1:100, bs-1189R; Bioss), mouse anti-Ezrin (1:300, sc-58758; Santa Cruz), rabbit anti-Zbtb7b (1:300, 11,341–1-AP; Proteintech) and mouse anti-E-cadherin (1:200, 610,182; BD Biosciences). FITC-conjugated UEAI lectin (1:2000, L32476; Thermo Fisher) and lectin GS-II conjugate with Alexa Fluor 647 (100 μg/mL, L-32451; Thermo Fisher).

### Human and mouse gastric organoid culture

The gastric tissue samples from human and mouse stomachs were thoroughly washed with cold phosphate-buffered saline (PBS) to remove contaminants. Subsequently, the tissues were incubated with 20 mM EDTA at 4 °C for 25 min. The resulting gastric glands from both the corpus and antrum were carefully scraped and collected, followed by centrifugation at 1000 rpm for 3 min. The gastric glands were then embedded in Matrigel (BD Biosciences) and seeded onto a 24-well plate. Once the Matrigel had solidified, culture medium was added. The basal culture medium contains Advanced DMEM/F12 supplemented with 2 mM GlutaMAX, 1 mM N-acetylcysteine, 1X N2, 1X B-27, and penicillin/streptomycin (all from Thermo Fisher). The complete medium (CM) for human and mouse stomach organoids consisted of the basal medium supplemented with 50 ng/mL EGF (epidermal growth factor), 100 ng/mL Noggin, 500 ng/mL R-spondin-1, 5 μM CHIR-99021, 10 nM gastrin, 100 ng/mL FGF10, 10 μM SB431542, and 10 μM Y-27632. To achieve cell differentiation, the following factors or molecules were used: BMP4 (20 ng/mL, R&D), LDN193189 (1 μM, MedChemExpress), Isx-9 (20 μM, Selleck), A83-01 (5 nM, Selleck), metformin (100 μM, Selleck), DY131 (5 μM, Selleck), PD0325901 (2 μM, Selleck), IXA4 (10 μM, MedChemExpress), IWP2 (2 μM, Selleck), Amphiregulin (100 ng/mL, ACROBiosystems), Neuregulin-1 (100 ng/mL, ACROBiosystems), and Wnt3a (100 ng/mL, ACROBiosystems). The organoids for cell differentiation were harvested 2 days after changing to differentiation media.

### Generation of Zbtb7b KO and Zbtb7b OE organoids

A lentiviral system was used to overexpress Zbtb7b in organoids. Lentivirus and recombinant adeno-associated virus (AAV) were produced following established protocols (Busslinger et al. 2021; Koo et al. 2011). Prior to virus infection, organoids were cultured in the complete medium supplemented with 10 mM nicotinamide for 2 days. Subsequently, the organoids were dissociated using TrypLE and resuspended in expansion medium supplemented with 10 mg/mL polybrene (Macgene, MC032) containing the virus. We seeded 250 μL of this medium with polybrene and virus onto pre-solidified Matrigel and incubated overnight at 37 °C. The following day, the medium was removed, and the virus was washed off with warm PBS. Then, 10 μL of Matrigel was overlaid, and the organoids were cultured in the complete medium. For lentivirus infection, the complete medium was changed to the medium containing 2 mg/mL puromycin 2 days post-infection to select for infected cells. Organoids were selected with puromycin and harvested for immunoblotting, ensuring the successful overexpression of Zbtb7b.

To achieve Zbtb7b knockout in organoids, HEK293 T cells were transfected with AAV carrying single-guide RNAs targeting exon 2 of Zbtb7b (5’-ATTCCCAGACCACAGC-3’). The organoids derived from *Cldn18*^*CreERT2*^*;Rosa26*^*loxp−stop−loxp−Cas9−EGFP*^ mice were pre-treated with 4-OHT to induce Cas9 expression. Zbtb7b knockout clones (KO1, KO2, and KO3) were screened by immunoblotting and confirmed by genomic DNA sequencing, with KO1 being utilized in subsequent studies.

### Immunoblotting

Cells were lysed in RIPA buffer (Beyotime, P0013B) supplemented with protease inhibitors (Roche, 04693132001) and PMSF (Beyotime, ST506). After quantification using a BCA protein assay kit (Beyotime, P0012S), 40 μg of total proteins were separated by 7.5% SDS-PAGE under denaturing conditions and transferred to nitrocellulose membranes (PALL, 66,485). The membranes were blocked and then incubated with primary antibodies overnight at 4 °C, followed by incubation with anti-rabbit or anti-mouse conjugated antibodies.

### Preparation of tissue samples and organoids for scRNA-seq

Gastric tissues were obtained from adult mouse and human for further analysis. Mouse stomach samples for scRNA-seq were collected from three wild-type mice, pooling corpus or antrum tissues by scraping gastric glands. Human stomach samples consisted of corpus tissues were from three individuals, and antrum tissues from four individuals, as detailed in Table S2. Gastric tissue fragments were incubated with a 20 mM EDTA digestion solution for 25 min at 4 °C. After incubation, gastric glands were scraped from the tissue and collected. To obtain a single-cell suspension, tissues were digested with TrypLE (Invitrogen) for 10~15 min at 37 °C. The mouse or human gastric organoids for scRNA-seq were generated from 6~8 individual wells of 24-well plates for specific differentiation media. Similarly, organoids were also digested using TrypLE. After digestion, the cells were filtered through a 40 μm sieve to obtain a single-cell suspension. Propidium iodide (PI; 5 μg/mL) staining was performed to identify and exclude dead cells. The PI-negative cells were then sorted by fluorescence-activated cell sorting (FACS) using a Beckman instrument. The sorted single cells were loaded onto a single-cell chip using the 10X Genomics Chromium Single Cell 3′ Solution. cDNA library was constructed from the single cells, and the library was sequenced using an Illumina Novaseq 6000 sequencer (Illumina, San Diego, CA, USA) with paired-end 150 bp reads.

### Immunostaining

Human and mouse stomach tissues or organoids were washed with cold PBS and fixed with 4% paraformaldehyde at room temperature overnight. Organoids were embedded in 5% agarose prior to paraffin embedding. Paraffin-embedded sections were then dewaxed in xylene and dehydrated using a graded series of alcohol. Antigen retrieval was carried out, followed by three washes in PBS. To block and permeabilize the sections, a solution containing 0.3% Triton X-100 and 5% BSA was applied for 1 h at room temperature. The sections were then incubated with primary antibodies overnight at 4 °C. Fluorescein-conjugated secondary antibodies (Life Technologies, diluted at 1:300) and 4',6-diamidino-2-phenylindole (DAPI) were then added and incubated for 1 h at room temperature. Images were acquired using an Olympus FV3000 laser scanning microscope.

### Transmission electron microscopy

Organoids or mouse stomach tissues were fixed in a solution containing 2.5% glutaraldehyde in phosphate-buffered saline (PBS) and subsequently post-fixed with 0.5% osmium tetroxide. To enhance contrast, the organoids were treated with uranyl acetate and tannic acid. Dehydration was carried out using a series of ethanol solutions with increasing concentrations. The dehydrated organoids were then infiltrated with Polybed (Polysciences) for embedding. Cut sections of the filters were arranged in flat embedding molds along with Polybed. After polymerization, the samples were sectioned to a thickness of 60 nm using a Leica UC7 ultramicrotome (Leica). These sections were further contrasted with lead citrate. Finally, the prepared samples were examined using a Hitachi HT7800 transmission electron microscope (HITACHI) for analysis.

### Acridine orange staining

Mouse organoids at passage 2 were cultured in complete medium for 8 days. Afterward, they were exposed to differentiation medium containing BMP4 and Isx-9 for 2 days. The organoids were pre-incubated with 100 μM histamine for 30 min. Subsequently, the organoids were digested with cell recovery buffer and then stained with 1 mM acridine orange for 15 min. Images were acquired using an Olympus FV3000 laser scanning microscope.

### FACS analysis

To identify Muc5ac^+^ pit cells in mouse gastric organoids, the organoids were first dissociated in TrypLE (Invitrogen) for 15 min at 37 °C and passed through a 40-μm cell strainer. The dissociated cells were then stained with anti-Muc5ac antibody on ice for 1 h, followed by labeling with a secondary antibody. The cells were analyzed using flow cytometry (CytoFLEX S, Beckman) to detect and quantify Muc5ac-positive pit cells.

### Cut&Tag analysis

The Cut&Tag assay was performed using the Novo NGS CUT&TAG4.0 Kit (Novoprotein, N259-YH01) following the manufacturer’s instruction. Tissues were from isolated mouse gastric glands, and gastric organoids from both control and *Bmpr1a* cKO mice. The organoids were preincubated with 20 ng/ml BMP4 for 2 days. Quality control statistics for the samples were obtained using the Fastp tool (v.0.22.0). Mapping to the reference mouse genome mm10 was carried out using bowtie2 (v.2.2.5), and peak calling was performed using MACS2 (v.2.2.6). Visualization of binding peaks was conducted using IGV software (v.2.10.0).

### Dual luciferase reporter assay

The promoter region of Zbtb7b was cloned into the pGL3-CMV firefly luciferase vector. HEK293 T cells were seeded and co-transfected with pGL3-CMV-Zbtb7b-promoter and a Renilla luciferase plasmid using Lipo2000 (Invitrogen, 11,668,019). The relative luciferase activity was measured 48 h later using the Dual-Luciferase Reporter Assay System and a Microplate Chemiluminescence Meter (Berthold, LB960).

### RNA extraction and quantitative RT-PCR

RNA was extracted from the organoids using the RNeasy Mini Kit (Qiagen) for subsequent qPCR or RNA sequencing analysis. The cDNA was synthesized using the 1 st Strand cDNA Synthesis Super Mix (Novoprotein). Real-time PCR reactions were performed in triplicate using the qPCR SuperMix Plus (Novoprotein) on a LightCycler 480 instrument (Roche). The primer sequences for the selected genes can be found in Table S3. All experiments were conducted with three independent biological replicates.

### scRNA-seq processing and filtering

The raw reads obtained from sequencing were aligned to the corresponding genomes of the species used in the study: GRCh38/hg38 for human and GRCm38/mm10 for mouse. The unique molecular identifiers (UMIs) were estimated using Cell Ranger software (v3.1.0) (Zheng et al. 2017). The aligned features were subsequently loaded and processed using the Seurat package (v4.0.2) (Hao et al. 2021)in R version 4.0.5. To filter out low-quality cells, cells were excluded if they expressed fewer than 200 genes or if more than 20% of their expressed genes were mitochondrial genes.

### scRNA-seq normalization and clustering

After performing data preprocessing and quality control, the Seurat package was used for data normalization. The NormalizeData function was applied, utilizing LogNormalize as the normalization method with a scale factor of 100,000 (scale.factor = 100,000). To identify variable genes, the FindVariableFeatures function was utilized. Integration of the scRNA-seq data from the human or mouse stomach tissue samples or gastric organoids were performed using the FindIntegrationAnchors function, which aligns and merges datasets from different sources. The scaled gene expression data were projected onto principal components (PCs), and the first 30 PCs capturing the most significant sources of variation were selected for further analysis. Non-linear dimensionality reduction was performed using Uniform Manifold Approximation and Projection (UMAP) to visualize and cluster cells based on gene expression similarities. Clustering was conducted using the FindNeighbors and FindClusters functions, which assign cells into distinct clusters based on their expression patterns. Marker genes specific to each cluster were identified using the FindAllMarkers function to find genes differentially expressed between clusters. To account for batch effects between the corpus and antrum scRNA-seq data, batch correction was performed using canonical correlation analysis (CCA). This method identifies linear combinations of features that are maximally correlated across different datasets, preserving the shared correlation structure. Finally, the data batches were pooled into a single object for downstream analyses, ensuring the shared structure is maintained across all samples (Mayer et al. 2018).

### scRNA-seq differential gene expression analysis

To identify the signature genes of each cell type, the Seurat package was used, specifically the'FindAllMarkers'and'FindMarkers'functions. The'FindMarkers'function was utilized to identify signature genes by comparing the cell type of interest with another specific group of cells.

### Screening for specific genes in tissues and organoids

We used “FindMarkers” of R package “Seurat” to identify differentially expressed genes between tissues and organoids. The parameters were set to min.pct ≥ 0.25 (genes have expression in at least 25% of cells), and the adjusted p-value < 0.05. Genes with log2 Foldchange ≥ 1 or log2 Foldchange ≤ −1 were regarded as differentially expressed ones between tissues and organoids.

### Spearman correlation of specific cell types between tissues and organoids

To identify representative genes for specific cell types, we employed the FindMarkers function in Seurat, which allowed us to filter the top 2000 genes. We then calculated the average expression of these genes, referred to as log1pAverageExpression, within the specific cell cluster of tissues or organoids. Subsequently, we utilized the average expression of these top 2000 genes to determine the Spearman correlation between the tissues and organoids.

### Screening for signaling response genes

We curated gene lists associated with the BMP, EGF, TGF-β, and Wnt signaling based on existing literature (Table S7). To determine cell types with the highest expression of these signaling pathways, we calculated the sum of gene expression for pathway-related genes within each cell type using the colSums function. This analysis enabled us to identify cell type-signaling pathway pairs. Subsequently, we selected the top 100 genes in specific cell types and calculated the correlation between the expression of each gene and the total expression of the corresponding signaling pathway in each cell. The genes exhibiting the highest correlation coefficients were designated as the response genes of the specific pathway (Table S8 and Table S10).

### RNA-seq analysis

Mouse corpus glands, mouse gastric organoids and human gastric organoids were harvested for bulk RNA sequencing. Bulk RNA-seq reads were aligned to the human or mouse genome (Ensembl release 95) using HISAT2 (v2.1.0) (Kim et al. 2019). SAM files were subsequently sorted and converted to BAM files using SAMtools (v1.9) (Li et al. 2009). Transcript assembly was performed using StringTie (v1.3.3b) (Frazee et al. 2015). Transcript expression levels were quantified, and quantitative gene expression (FPKM) was obtained using Ballgown (v2.16.0) (Frazee et al. 2015; Pertea et al. 2016). Genes with FPKM less than 1 were considered unexpressed and excluded. Differential expression analysis was performed by identifying genes with |log2 FC| greater than 1 and P < 0.05. Functional enrichment analysis was conducted using the DAVID online software tool (https://david.ncifcrf.gov) with default parameters.

### Statistics

The images were processed using Adobe Photoshop CC (2017). Statistical analyses were conducted using Prism (v8). To determine statistically significant differences, ordinary two-way ANOVA followed by Tukey's multiple comparison test and unpaired multiple t-test was employed. n.s. (not significant), * (*p* < 0.05), ** (*p* < 0.01), *** (*p* < 0.001), and **** (*p* < 0.0001).

## Supplementary Information

Supplementary Material 1: Supplementary figures 1-14

Supplementary Material 2: Supplementary tables 1-11

## Data Availability

The RNA-seq data, scRNA-seq data and cut&tag data generated in this study are publicly available through the Gene Expression Omnibus (GEO) with the accession code GSE226201. All other data are available from the corresponding author on request.

## Abbreviations

BMP4: Bone morphogenetic protein 4
EEC: Enteroendocrine cell
EGF: Epidermal growth factor
TGF-β: Transforming growth factor beta
Zbtb7b: Zinc finger and BTB domain containing 7B
GSII: Griffonia simplicifolia lectin II
UEAI: Ulex Europaeus Agglutinin I
ATP4B: ATPase H + /K + Transporting Subunit Beta
AREG: Amphiregulin
NRG1: Neuregulin 1
scRNA-seq: Single cell RNA sequencing
UMAP: Uniform Manifold Approximation and Projection
Cut&Tag: Cleavage Under Targets and Tagmentation
ChIP-qPCR: Quantitative real-time PCR after Chromatin Immunoprecipitation

## Code availability

All codes that enable the main steps of the analysis are available from the corresponding author under request.
