# Supplementary material for: The critical role of BMP signaling in gastric epithelial cell differentiation revealed by organoids
Source: Cell Regen. 2025 May 16;14:18. doi: 10.1186/s13619-025-00237-x (PMC12084451; doi:10.1186/s13619-025-00237-x)
Supplement: Supplementary file 1 — Supplementary Material 1: Supplementary figures 1-14 [file 13619_2025_237_MOESM1_ESM.docx]

Supplemental Materials

**The critical role of BMP signaling in gastric epithelial cell differentiation revealed by organoids**

Fan Hong^1#^, Xiaodan Wang^2#&^, Nanshan Zhong^3^, Ze Zhang^1^, Shibo Lin^4^, Mengxian Zhang^2^, Haonan Li^2^, Yuan Liu^2^, Yalong Wang^1^, Lianzheng Zhao^2^, Xiao Yang^5^, Hongwen Zhou^6^, Hui Liang^4^, Ye-Guang Chen^1,2,3*^

Supplementary figures: S1-S14

**
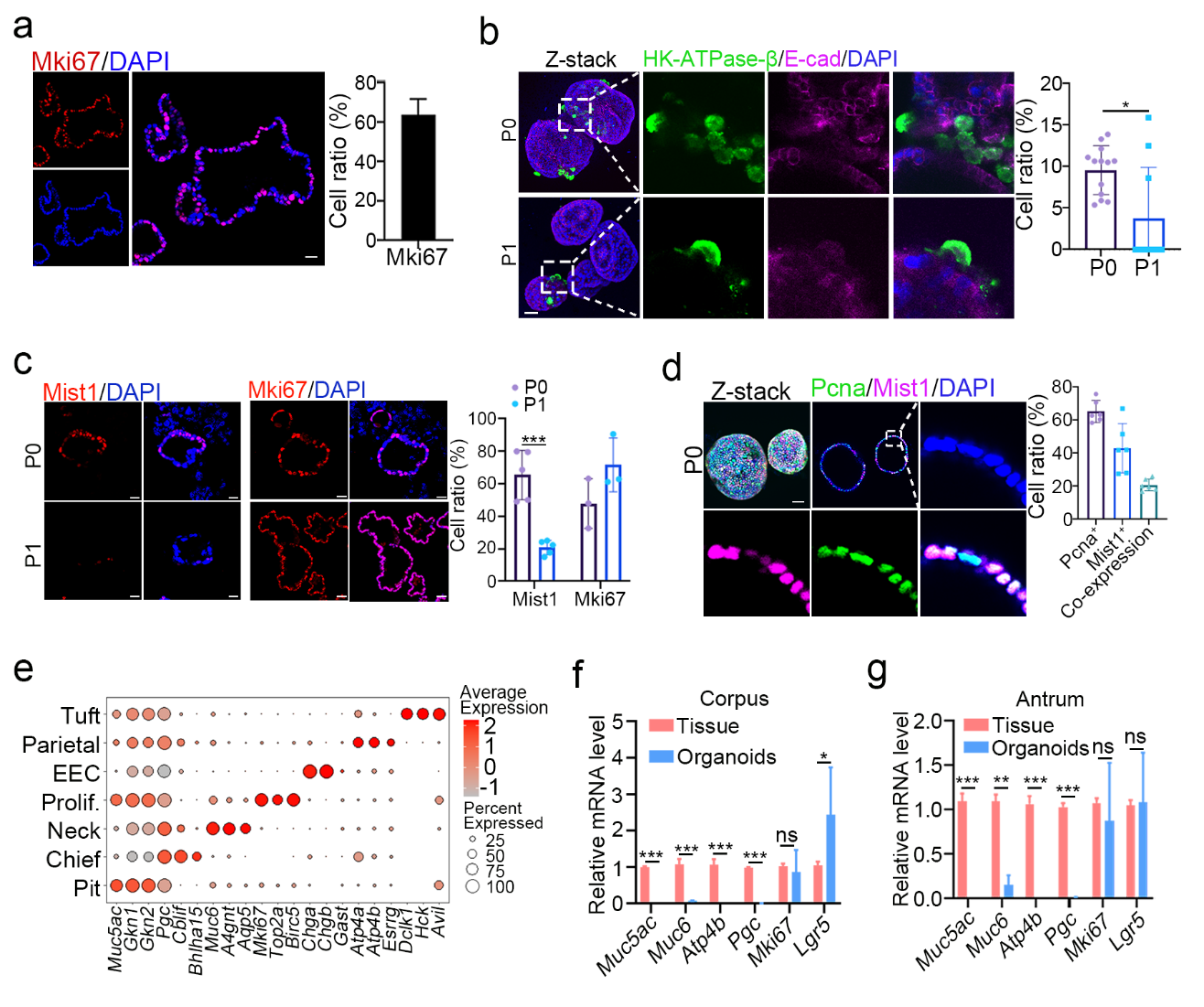
**

**Figure S1. The cell types in the organoids cultured in the complete medium, related to Figure 1.**

a, Immunofluorescent staining and quantification of mouse gastric organoids cultured with the complete medium for Mki67 (n=3). Scale bar: 20 μm. b-c, Immunofluorescent staining of HK-ATPase-β, Mist1, Mki67 and E-cad, and quantification in mouse gastric organoids cultured with the complete medium at passage 0 or passage 1. Scale bars, 50 μm in (b) and 20 μm in (c). b: z-stack merged views with enlarged images. d, Immunofluorescent staining and quantification of mouse gastric organoids at P0 cultured with the complete medium for Pcna and Mist1 (n=5). Scale bar: 20 μm. Z-stack merged views with enlarged images. e, Dot plot of cell lineage markers related to different types of gastric epithelial cells in Fig. 1d. f-g, Relative mRNA expression levels of gastric cell lineage markers in mouse corpus and antrum organoids compared to their corresponding tissues. All immunofluorescence images were counter-stained with DAPI to show nuclei. Statistical significances were determined by unpaired multiple t test. *p < 0.05, **p < 0.01, ***p < 0.001.

**
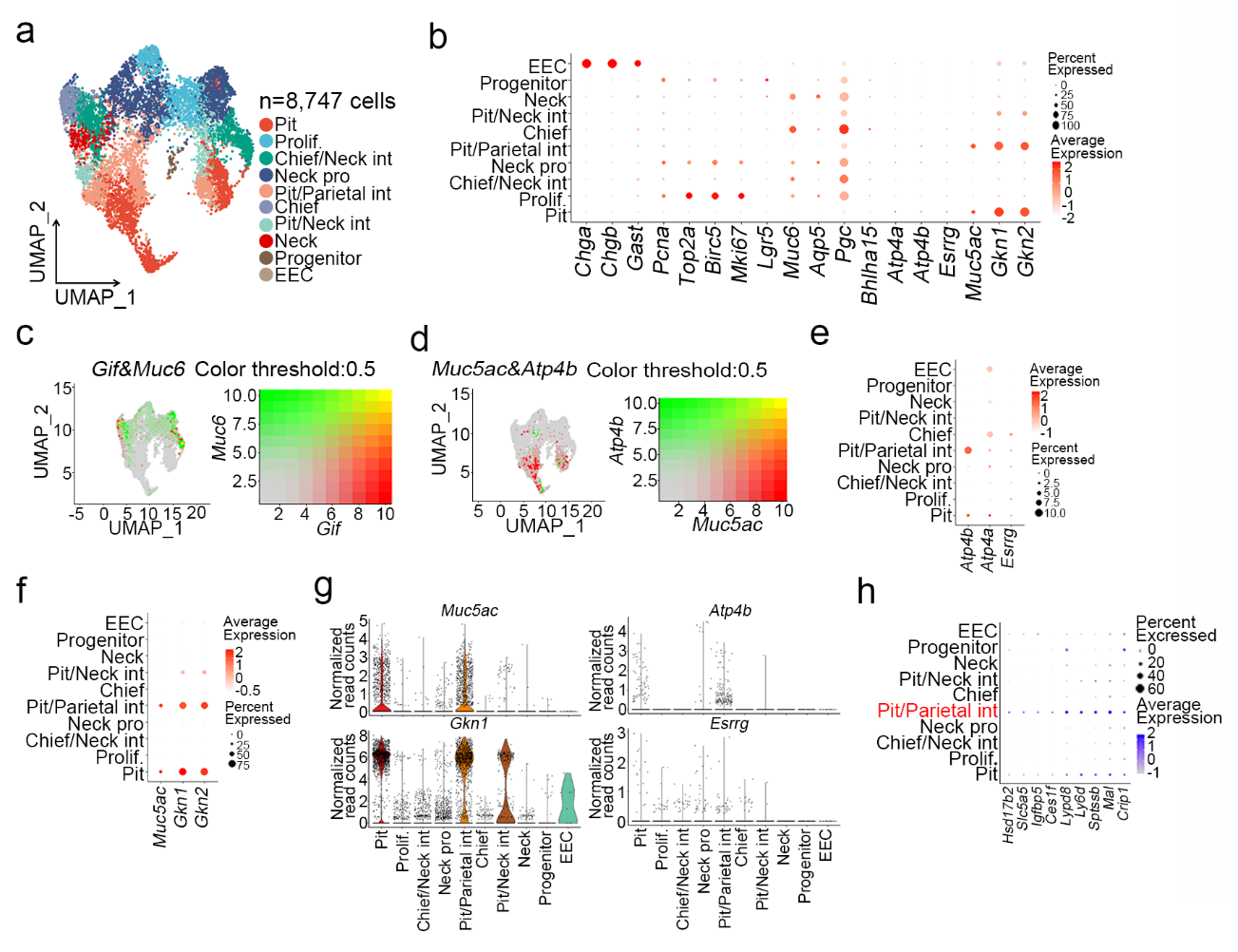
**

**Figure S2. ScRNA-seq analysis of mouse organoids, related to Figure 1.**

a, UMAP visualization of 10 single-cell clusters comprising 8,747 cells from mouse gastric organoids (n=4). Prolif.: Proliferative cell; Pit/Parietal int: Pit and Parietal intermediates; Pit/Neck int: Pit and Neck intermediates; Chief/Neck int: Chief and Neck intermediates; Neck: neck mucous cells; Neck pro: Neck progenitor; EEC: enteroendocrine cells. b, Dot plot of lineage markers of gastric epithelial cells in Fig. S2a. c-d, UMAP plots showing co-expression of *Gif* and *Muc6*, *Muc5ac* and *Atp4b* in each cluster of mouse gastric organoids. The Y-axis indicates the expression levels of *Muc6* (c) or *Atp4b* (d), with the color gradient from grey to green representing expression levels from low to high. The X-axis represents the expression of *Gif* (c) and *Muc5ac* (d), with the color gradient from grey to red indicating expression levels from low to high. Cells co-expressing *Muc6* and *Gif* (c) or *Atp4b* and *Muc5ac* (d) are shown in yellow. e-f, Dot plots showing the expression levels of the markers of parietal cells (e) and pit cells (f). g, Violin plot showing normalized read counts of the lineage markers of parietal cells (*Atp4b, Esrrg*) or pit cells (*Muc5ac, Gkn1*). h, Dot plot of signature genes related to pit/parietal intermediate cells.

**
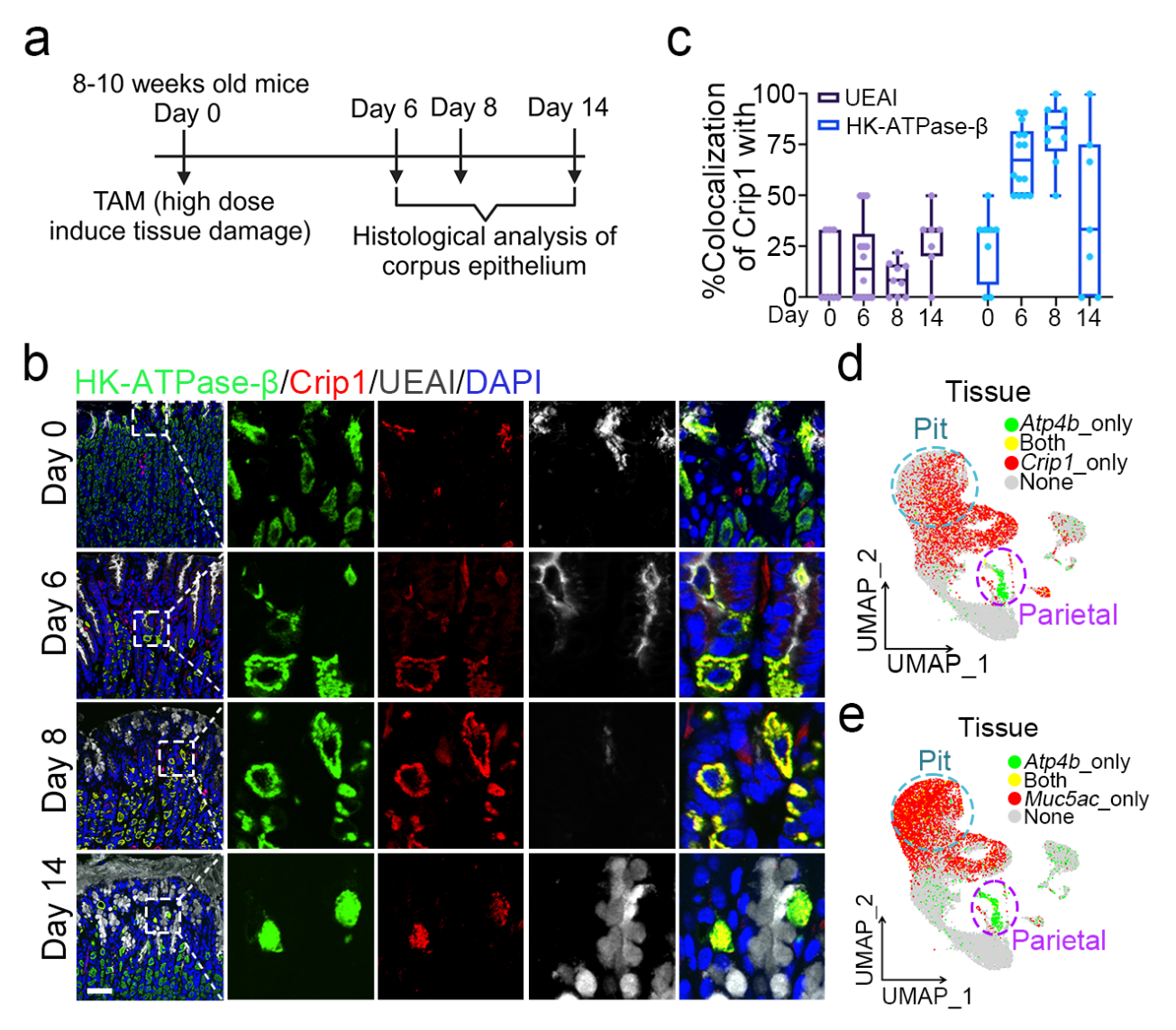
**

**Figure S3.** **Identified pit/parietal intermediate cells in vivo, related to Figure 1.**

a, Experimental strategy using a high dose of tamoxifen (40 mg/ml, 100 μl single injection) to induce tissue damage over a 2-week period in 8-10 week-old wild-type mice. b-c, Immunofluorescent staining and quantification of corpus gland of wild type mice for HK-ATPase-β, Crip1 and UEAI. Scale bars, 100 μm. d-e, UMAP plots showing co-expression of *Atp4b* and *Crip1* (d) and co-expression of *Muc5ac* and *Atp4b* (e) in each cluster of scRNA-seq data from mouse gastric tissues. Green indicates exclusive expression of *Atp4b* (d-e), red indicates exclusive expression of *Crip1* (d) or *Muc5ac* (e), yellow indicates co-expression of both *Atp4b* and *Crip1* (d) or both *Atp4b* and *Muc5ac* (e), and grey indicates no expression of these genes.

**
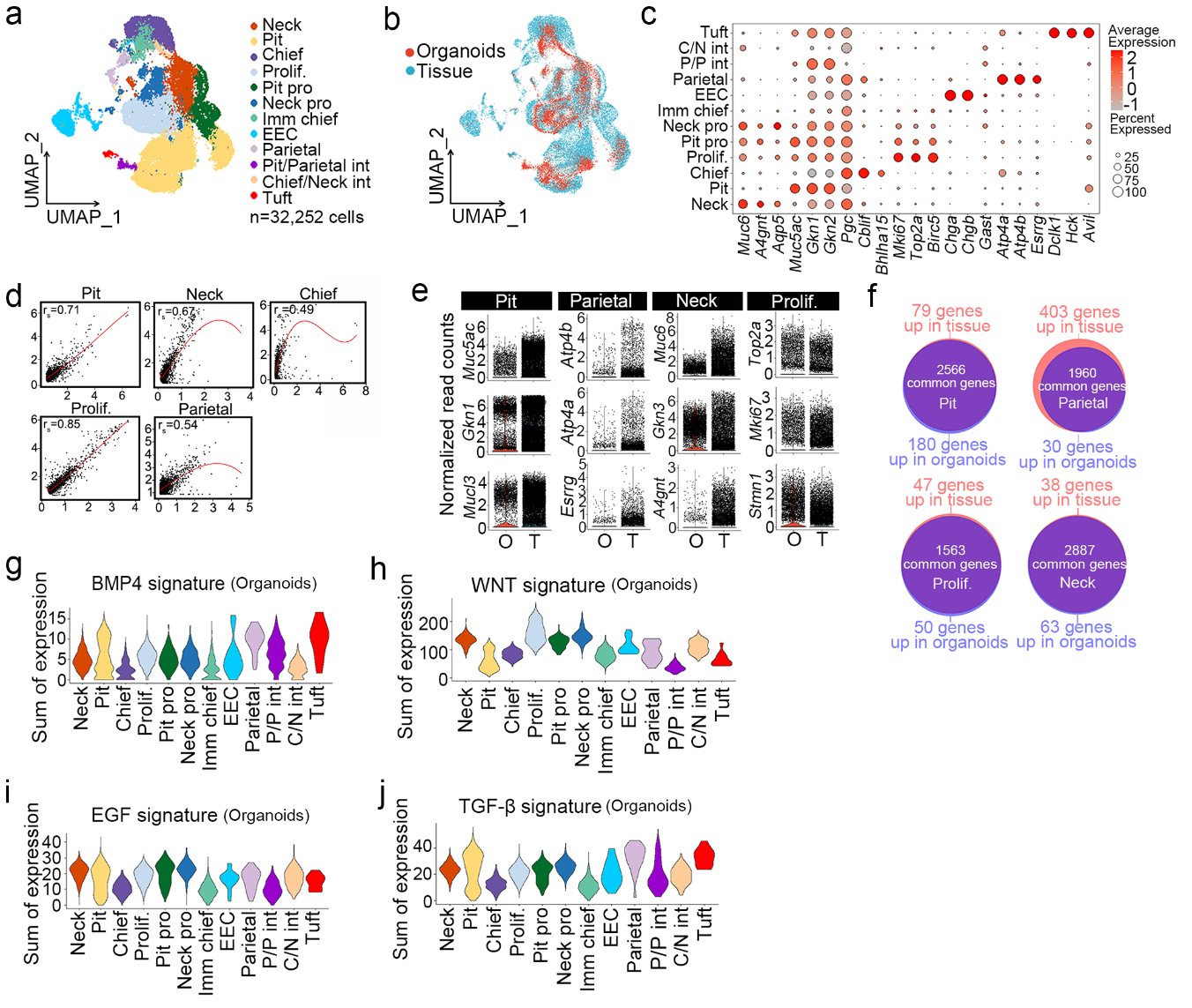
**

**Figure S4. ScRNA-seq analysis of both mouse stomach tissues and organoids, related to Figure 1.**

a, UMAP visualization of 12 single-cell clusters comprising 32,252 cells from mouse stomach epithelial tissue (n=4) and organoids (n=4). b, UMAP plots for mouse tissue (light blue) and gastric organoids cells (red). c, Dot plot of lineage markers of gastric epithelial cells in Fig. S4a. d, The Spearman correlation analysis of scRNA-seq expression (UMI count) of characteristic genes for pit cell, neck cell, proliferative cell, parietal cell and chief cell in tissues (X axis) and organoids (Y axis). Rs: R-squared score. e, Violin plot with scatter points showing normalized read counts of cell lineage markers of pit, parietal, neck or proliferative cells. f, Venn diagram showing the number of commonly and differentially expressed genes between tissue and organoids-derived cells by scRNA-seq; log2 Foldchange ≥ 1 or log2 Foldchange ≤ -1, p < 0.05. g-j, Violin plot showing overall expression of BMP4 signature (g), WNT signature (h), EGF signature (i) and TGF-β signature (j) in each cell cluster of mouse gastric organoids.

**
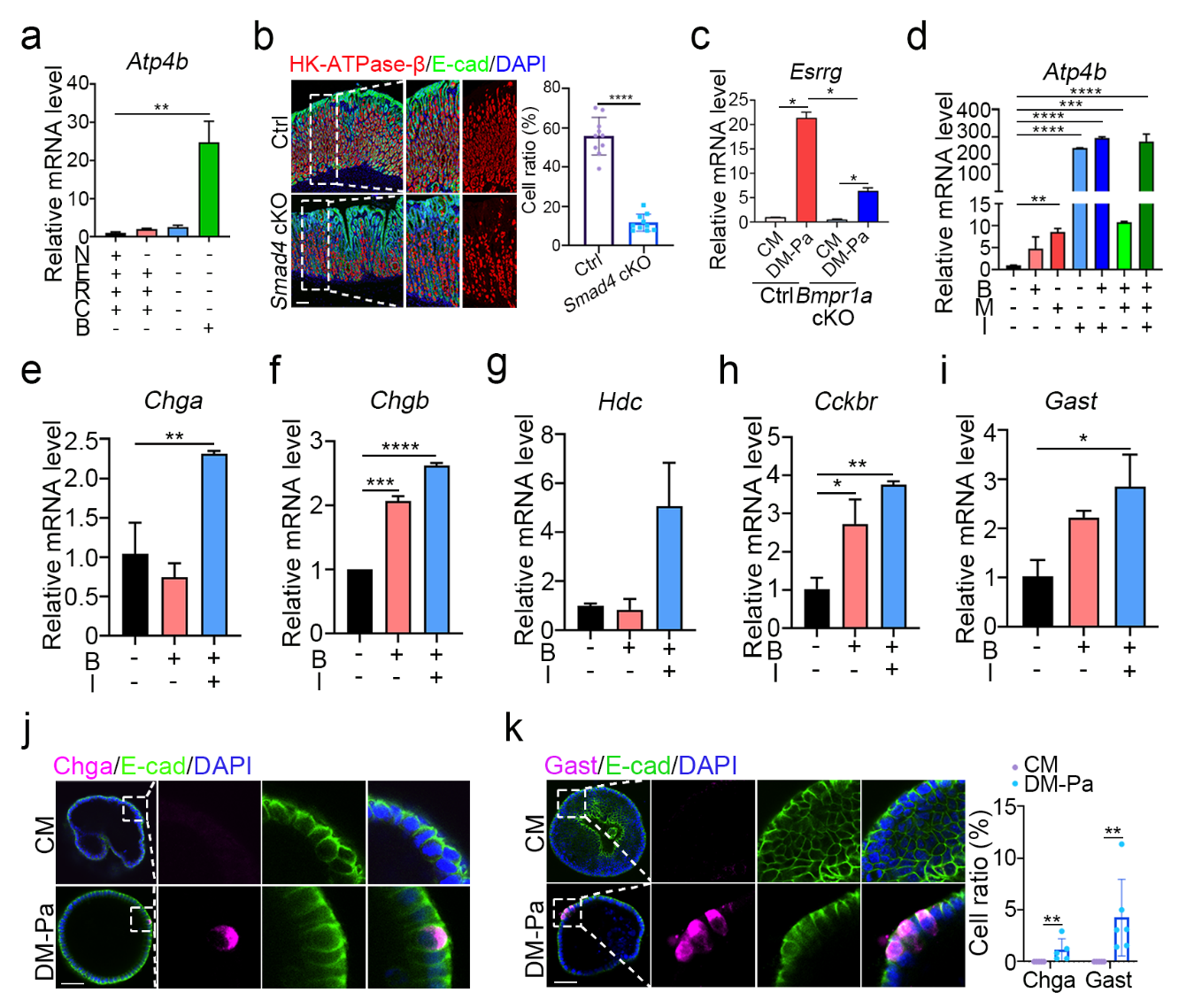
**

**Figure S5. BMP4 promotes the differentiation of parietal and enteroendocrine cells, related to Figure 2.**

a, The mRNA expression of *Atp4b* in mouse gastric organoids cultured under the indicated conditions for 2 days. N: Noggin, E: EGF, R: Rspondin1, C: CHIR-99021, B: BMP4. b, Immunofluorescent staining of HK-ATPase-β and E-cad, and quantification in corpus glands of *Smad4* cKO mice (n=3). Scale bars, 100 μm. c-i, The mRNA expression of *Esrrg*, *Atp4b*, *Chga*, *Chgb*, *Hdc,* *Cckbr* and *Gast* in mouse gastric organoids cultured under the indicated conditions for 2 days. B: BMP4, M: metformin and I: Isx-9. j-k, Immunofluorescent staining of Chga, Gast and E-cad, and quantification in mouse gastric organoids cultured under the indicated conditions (n=5). Scale bars, 50 μm. All immunofluorescence images were counter-stained with DAPI to show nuclei. Statistical significances were determined by unpaired multiple t test. *p < 0.05, **p < 0.01, ***p < 0.001, ****p < 0.0001.

**
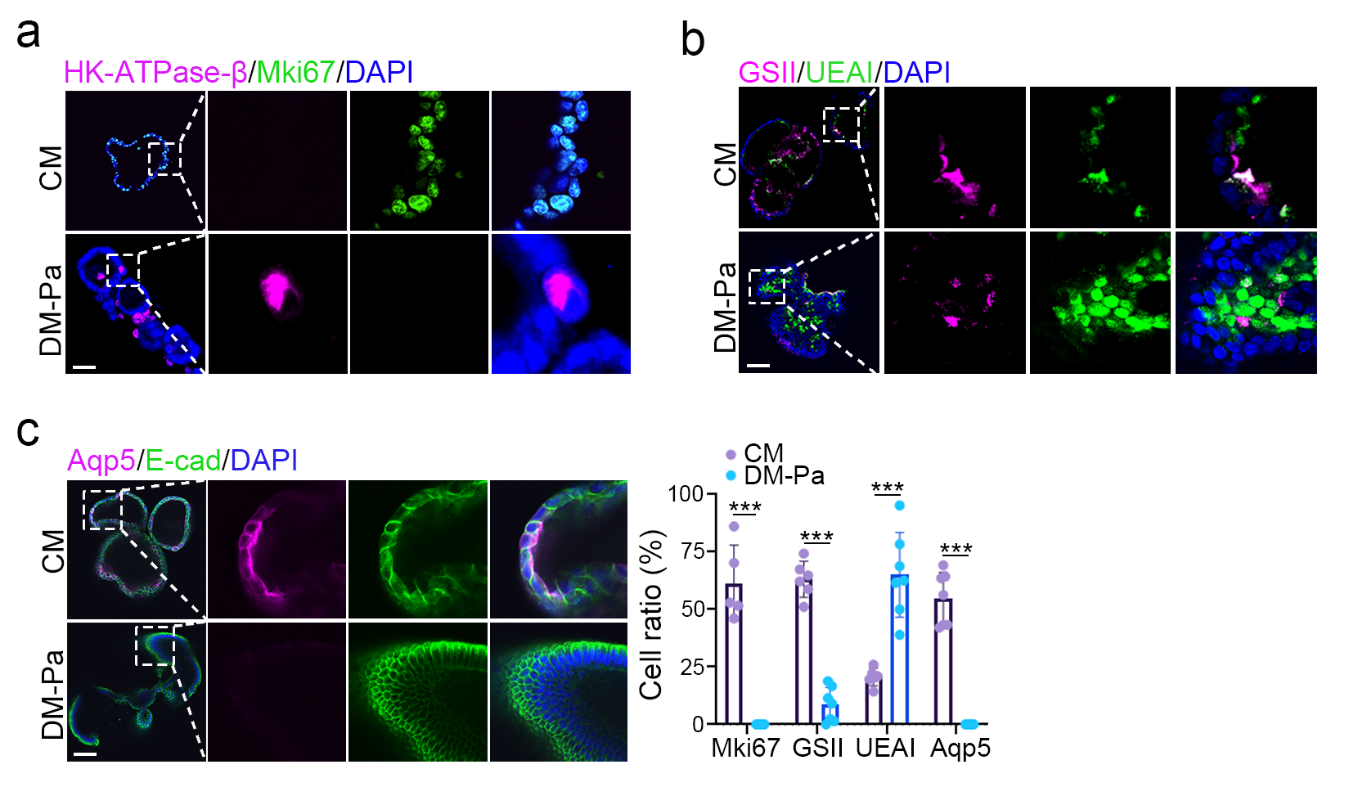
**

**Figure S6. Differentiation of parietal cells and enteroendocrine cells in the DM-Pa medium, related to Figure 2.**

a-c, Immunofluorescent staining of HK-ATPase-β, Mki67, GSII, UEAI, Aqp5 and E-cad, and quantification in mouse gastric organoids cultured under the indicated conditions (n=6). Scale bars, 50 μm. All immunofluorescence images were counter-stained with DAPI to show the nuclei. Statistical significances were determined by unpaired multiple t test. ***p < 0.001.

**
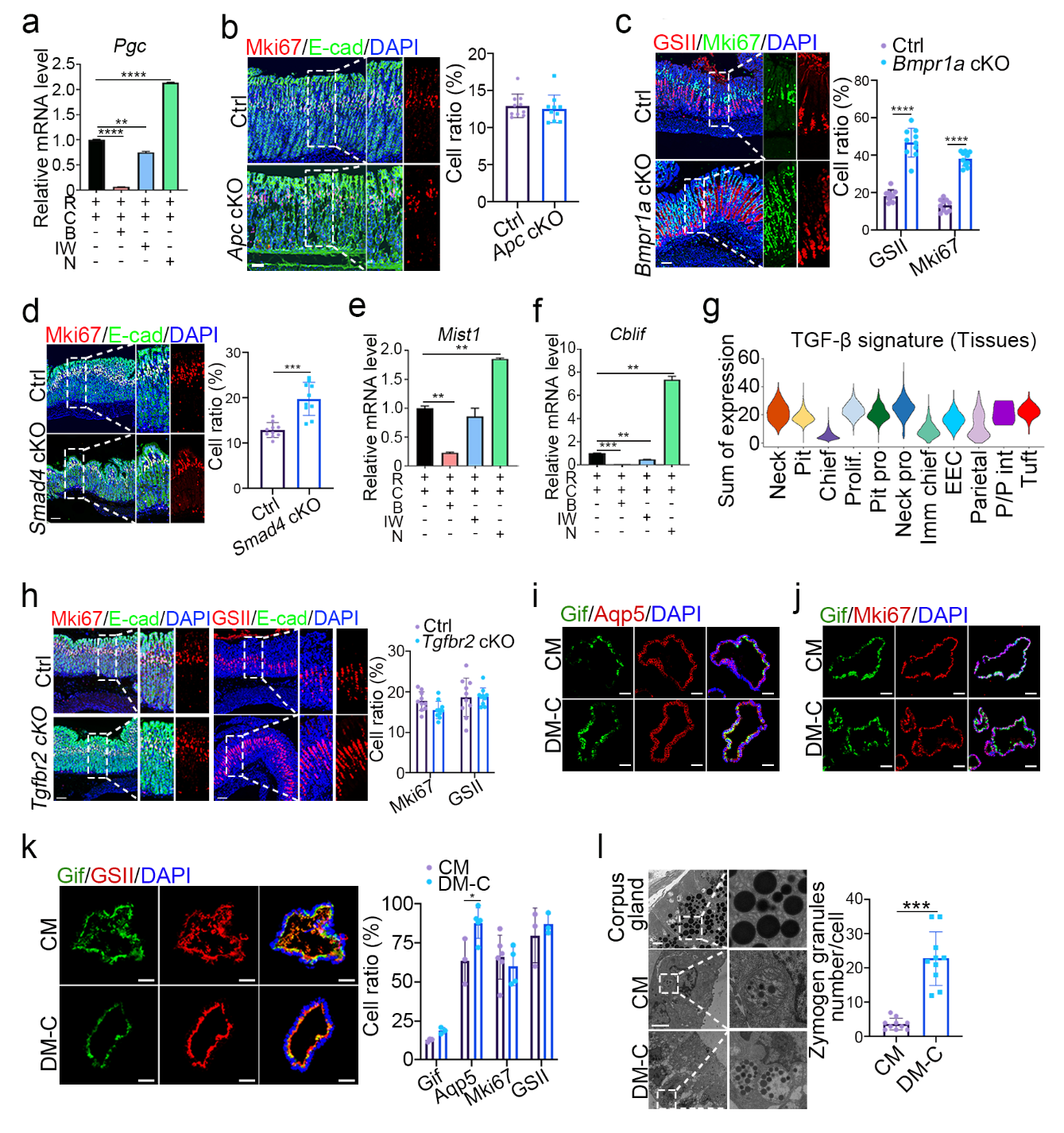
**

**Figure S7. Wnt and TGF-β signaling regulate the differentiation of chief cell, related to Figure 3.**

a, The mRNA expression of *Pgc* in mouse gastric organoids cultured under the indicated conditions for 2 day. R: Rspondin1, C: CHIR-99021, B: BMP4, IW: IWP2, N: Noggin. b-d, Immunofluorescent staining of Mki67, GSII and E-cad and quantification in the corpus region of control, *Apc* (b), *Bmpr1a* (c) or *Smad4* (d) cKO mice (n=3). Scale bars, 100 μm. e-f, The mRNA expression of *Mist1* and *Cblif* in mouse gastric organoids cultured under the indicated conditions for 2 days. g, Violin plot showing the expression of TGF-β signature in each cell cluster of mouse stomach tissue. h-k, Immunofluorescent staining of Mki67, GSII, Gif, Aqp5 and E-cad and quantification in corpus glands (h) or mouse gastric organoids (i-k) cultured under the indicated conditions (n=3). Scale bars in (h) is 100 μm, in (i-k) is 50 μm. l, Transmission electron microscope images, and quantification of zymogen granules numbers in mouse corpus gland and gastric organoids cultured under the indicated conditions for 2 days (n=10). Enlarged images highlight organelle details within cells. Scale bars: 2 μm. All immunofluorescence images were counter-stained with DAPI to show nuclei. Statistical significances were determined by unpaired multiple t test. *p < 0.05, **p < 0.01, ***p < 0.001, ****p < 0.0001.

**
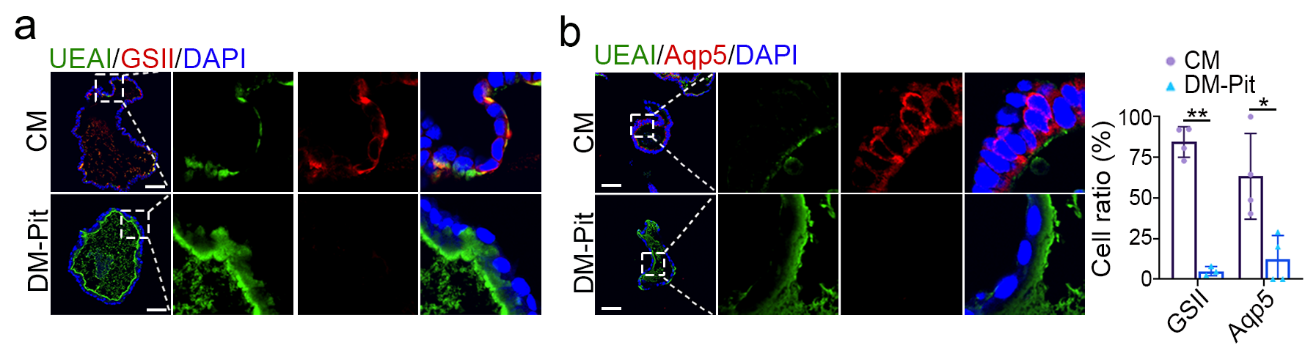
**

**Figure S8. Pit cell differentiation is enhanced by BMP4 and EGF signaling, related to Figure 4.**

a-b, Immunofluorescent staining of UEAI, GSII and Aqp5 and quantification of mouse gastric organoids cultured under the indicated conditions (n=3). Scale bars, 50 μm. All immunofluorescence images were counter-stained with DAPI to show nuclei. Statistical significances were determined by unpaired multiple t test. *p < 0.05, **p < 0.01.

**
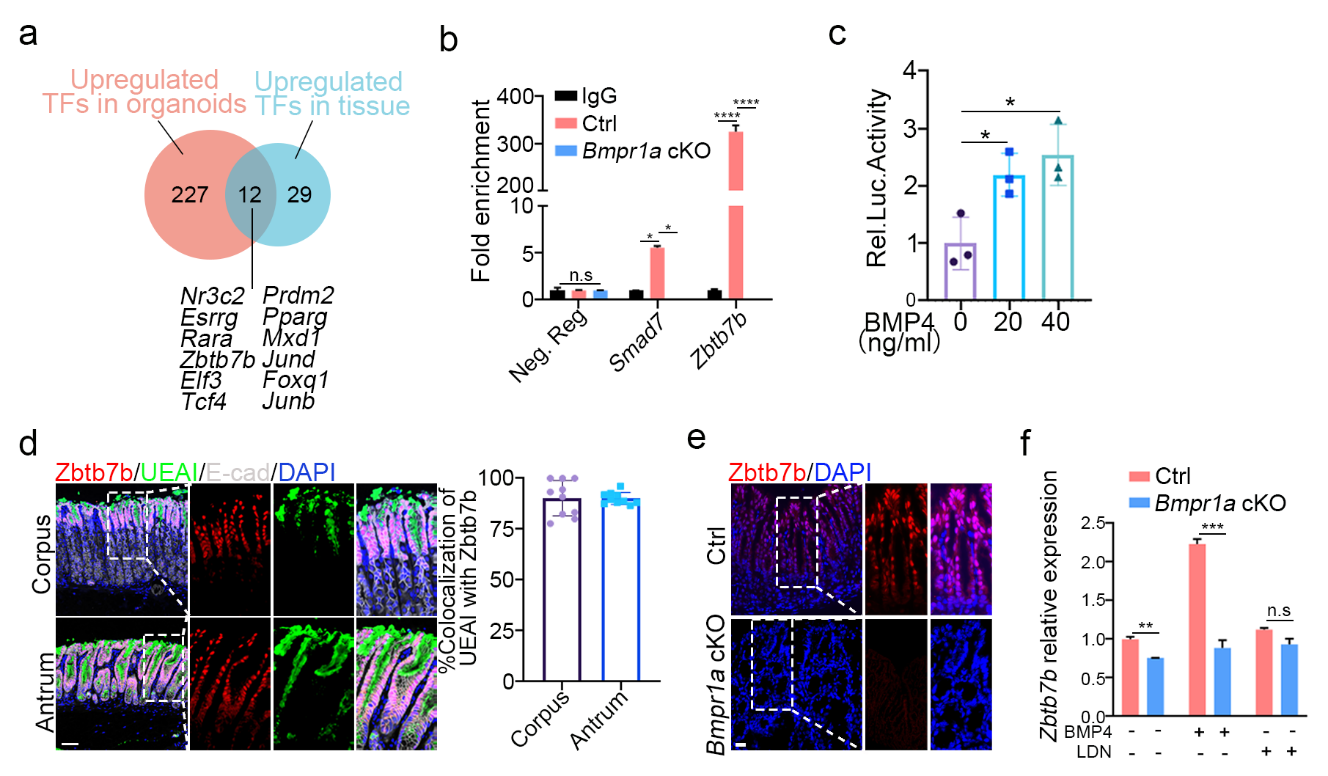
**

**Figure S9. Identification of transcriptional factors promoting pit cell differentiation, related to Figure 5.**

a, Venn diagram depicting transcription factors upregulated during pit cell differentiation in both mouse gastric tissues and organoids. b, Q-PCR followed Cut&Tag shows the enrichment of Smad1 in the promoter regions of indicated genes. ChIP-qPCR primers include in Table S3. c, HEK293T cells were transfected with Zbtb7b luciferase reporter, as well as with Renilla luciferase, then treated with indicated amounts of BMP4, and luciferase activity was determined 48 h later. d, Immunofluorescent staining of Zbtb7b, UEAI and E-cad and quantification in corpus and antral glands in wild type mice (n=3). Scale bars, 100 μm. e, Immunofluorescent staining of Zbtb7b in antrum glands of control or *Bmpr1a* cKO mice. Scale bars, 100 μm. f, mRNA expression levels of *Zbtb7b* in control or *Bmpr1a* cKO mouse gastric organoids, cultured for 2 days under the indicated conditions (BMP4: 20 ng/ml, LDN: LDN-193198, 1 μM). All immunofluorescence images were counter-stained with DAPI to show nuclei. Statistical significances were determined by unpaired multiple t test. *p < 0.05, **p < 0.01, ***p < 0.001, ****p < 0.0001.


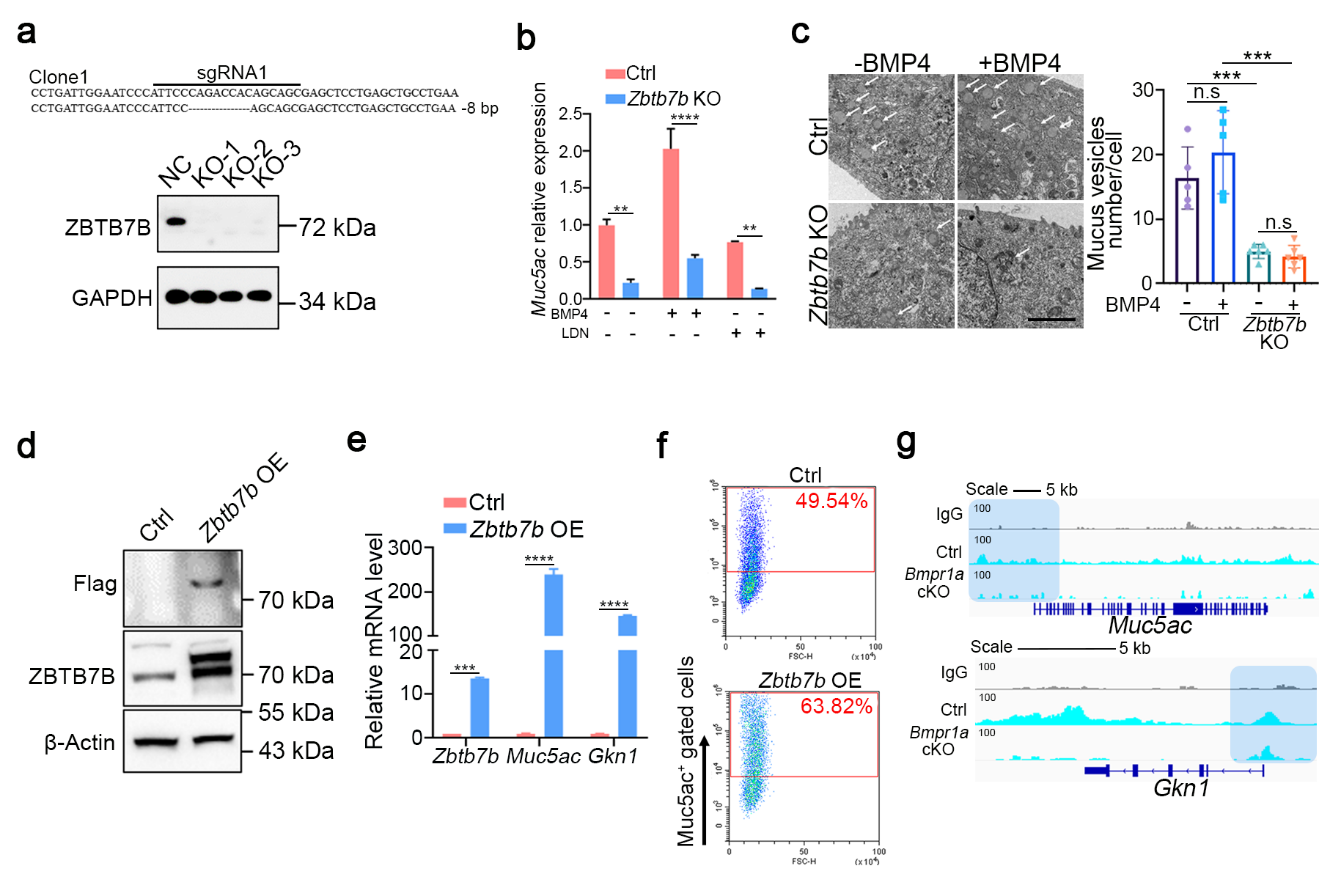


**Figure S10. Zbtb7b mediates pit cell differentiation, related to Figure 5.**

a, *Zbtb7b* KO gastric organoids was carried out with CRISPR/Cas9 using single-guide RNA 5’-ATTCCCAGACCACAGC-3’ targeting the exon 2 region of *Zbtb7b*. The efficiency of *Zbtb7b* KO was examined by immunoblotting. KO1 is a KO clones used in this study. b, mRNA expression levels of *Muc5ac* in mouse gastric organoids of control or *Zbtb7b* KO cultured for 2 days under the indicated conditions (BMP4: 20 ng/ml, LDN: LDN-193198, 1 μM). c, Transmission electron microscopy images and quantification of mucus vesicle numbers in control or *Zbtb7b* KO mouse gastric organoids, cultured for 2 days under specified conditions (n=5). BMP4: 20 ng/ml. d, Zbtb7b overexpression was examined by immunoblotting. e, The mRNA expression of lineage markers in control or Zbtb7b OE mouse gastric organoids cultured under the indicated conditions for 2 days. f, Fluorescence-activated cell sorting (FACS) analysis and quantitation of Muc5ac^+^ cells in control or Zbtb7b OE organoids. g, Cut&Tag analysis displaying Zbtb7b binding peaks on the *Muc5ac* and *Gkn1* promoter in control and *Bmpr1a* cKO stomach tissue. Statistical significances were determined by unpaired multiple t test. **p < 0.01, ***p < 0.001, ****p < 0.0001.


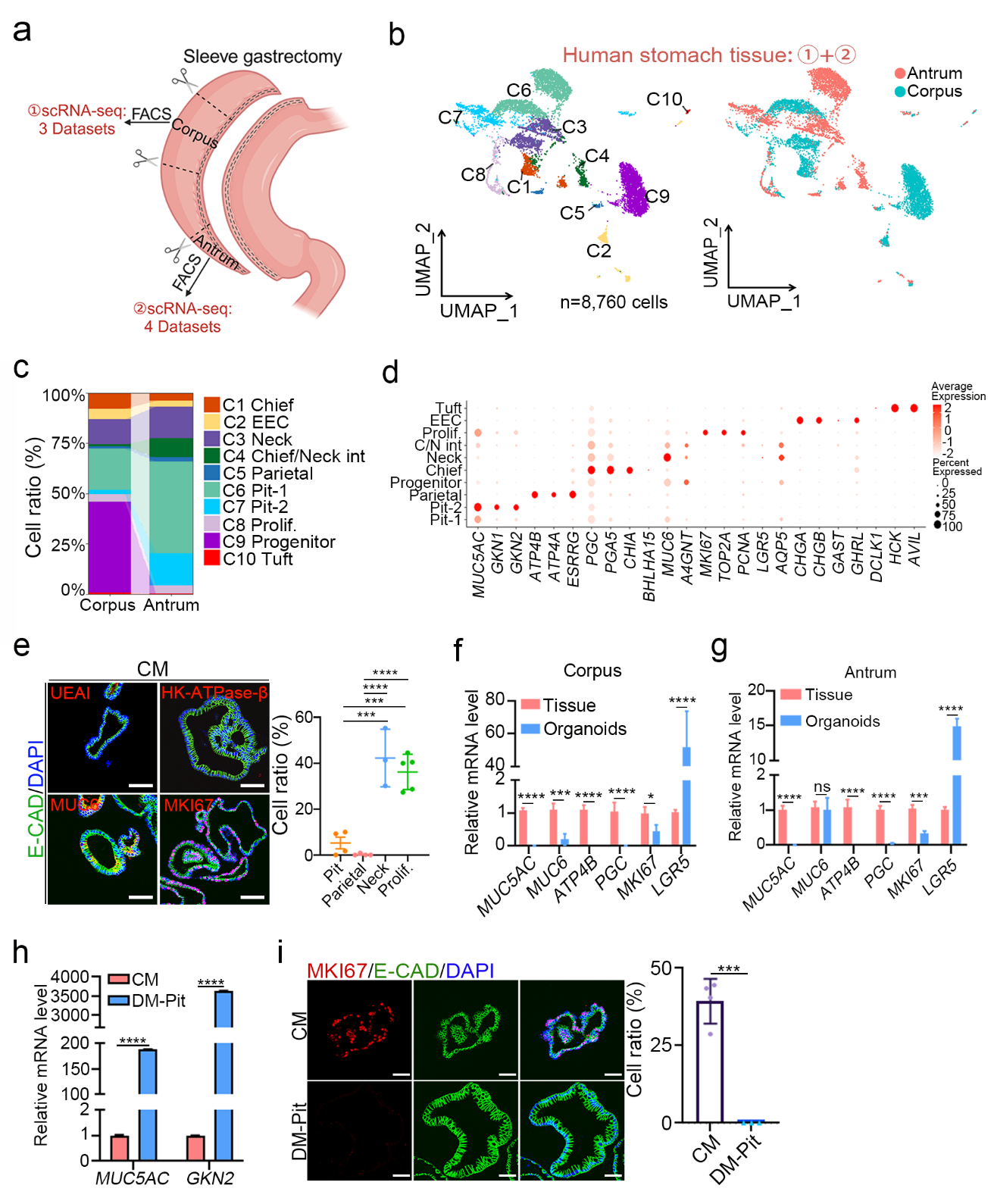


**Figure S11. Cell identity in human stomach tissue and organoids revealed by scRNA-seq, related to Figure 6.**

a, A schematic illustration of human stomach tissues derived from patients who underwent sleeve gastrectomy for scRNA-seq. b-c, UMAP visualization of 10 single-cell clusters comprising 8,760 cells from human corpus (n=3) and antral (n=4) epithelial cells and corresponding regional annotations (right panel), along with corresponding cell ratio (c). C1: Chief cell; C2: Enteroendocrine cell; C3: Neck mucous cell; C4: Chief/Neck intermediates; C5: Parietal cell; C6: Pit cell-1; C7: Pit cell-2; C8: Proliferative cell; C9: Progenitor; C10: Tuft. d, Dot plot of cell lineage markers of different types of gastric epithelial cells in (b). e, Immunofluorescent staining of UEAI, HK-ATPase-β, MUC6, MKI67 and E-CAD and quantification of human gastric organoids cultured under the complete medium (n=3). Scale bar: 50 μm. f-g, The mRNA expression of gastric cell lineage markers in human corpus (f) or antrum (g) organoids and corresponding tissue. h, The mRNA expression of pit cell markers in human gastric organoids cultured in the indicated medium for 2 days. i, Immunofluorescent staining of MKI67 and E-CAD and quantification of human gastric organoids cultured under the indicated medium (n=4). Scale bar: 50 μm. All immunofluorescence images were counter-stained with DAPI to show nuclei. Statistical significances were determined by unpaired multiple t test. *p < 0.05, ***p < 0.001, ****p < 0.0001.

**
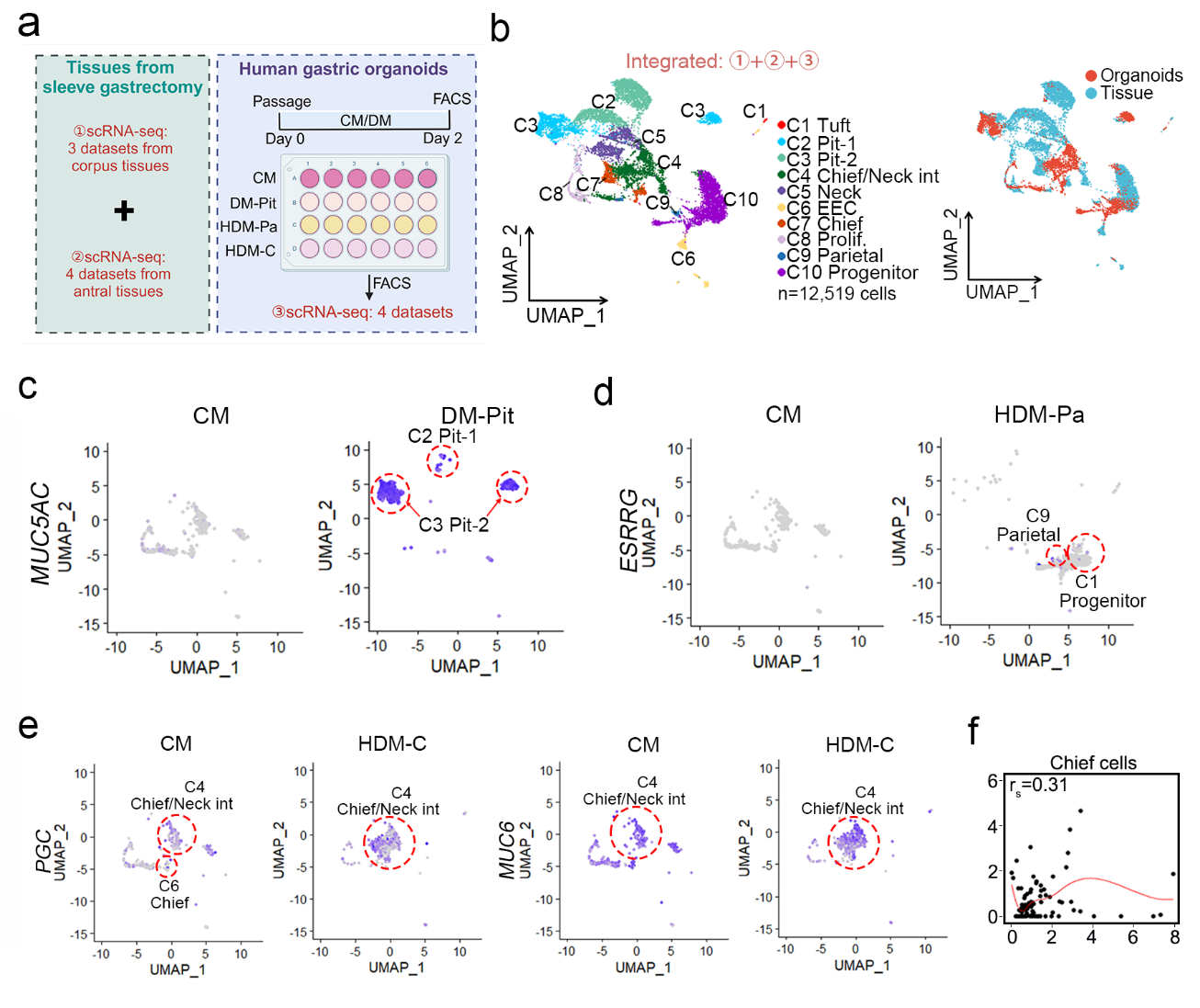
**

**Figure S12. Cell identity and clustering in human stomach tissue and organoids revealed by scRNA-seq, related to Figure 6.**

a, Human stomach tissues derived from patients who underwent sleeve gastrectomy and human gastric organoids were cultured in different culture media for scRNA-seq. b, UMAP visualization of 10 single-cell clusters comprising 12,519 cells from human stomach tissue (n=7) and gastric organoids (n=4). c-e, Feature plots show the expression of the cell lineage markers *MUC5AC* (c), *ESRRG* (d), *PGC* and *MUC6* (e) in human gastric organoids cultured in the indicated media. f, The Spearman correlation analysis of scRNA-seq expression (UMI count) of characteristic genes for chief cell in human gastric tissues (X axis) and organoids (Y axis). Rs: R-squared score.

**
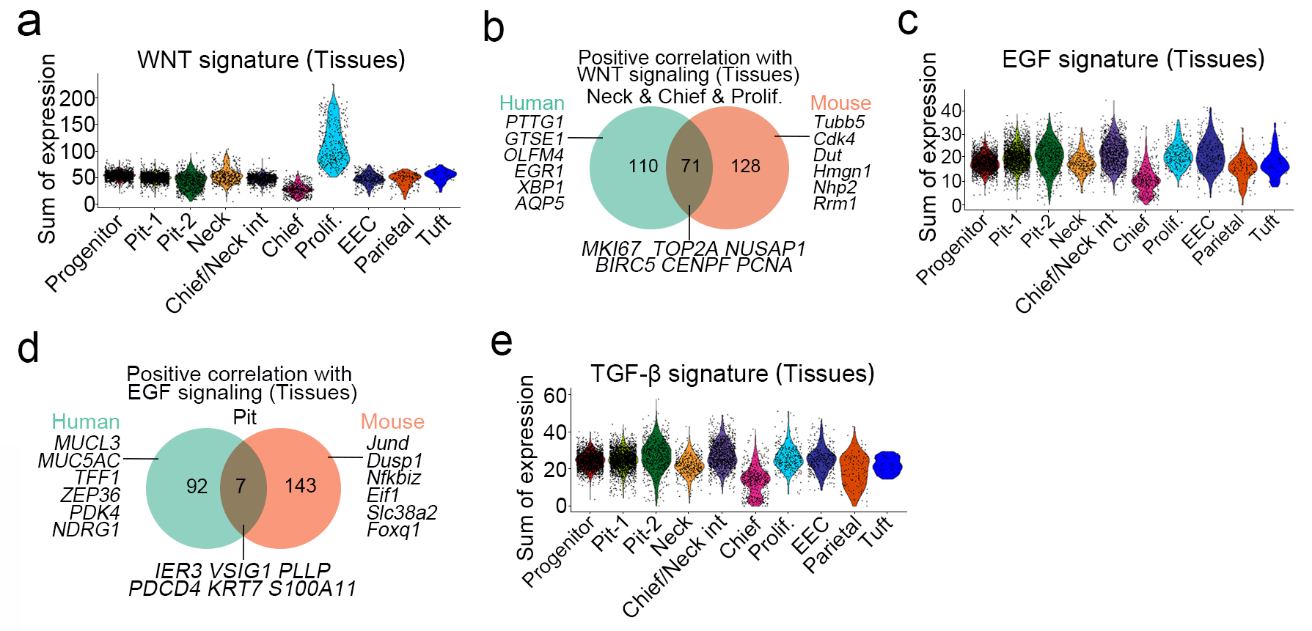
**

**Figure S13. Signature gene expression of different signaling pathways in human stomach tissue and organoids revealed by scRNA-seq, related to Figure 6.**

a, c, e, Violin plot showing the expression of WNT (a), EGF (c), TGF-β (e) signature in different clusters of human gastric epithelial cells in vivo. b, Venn diagrams displaying genes in proliferative cell, neck cell and chief cell lineages positively correlated with Wnt signaling in human stomach tissue. d, Venn diagrams displaying genes in pit cell lineages positively correlated with EGF signaling in human stomach tissue. Left: Human-specific genes. Middle: Genes conserved in human and mouse. Right: Mouse-specific genes.

**
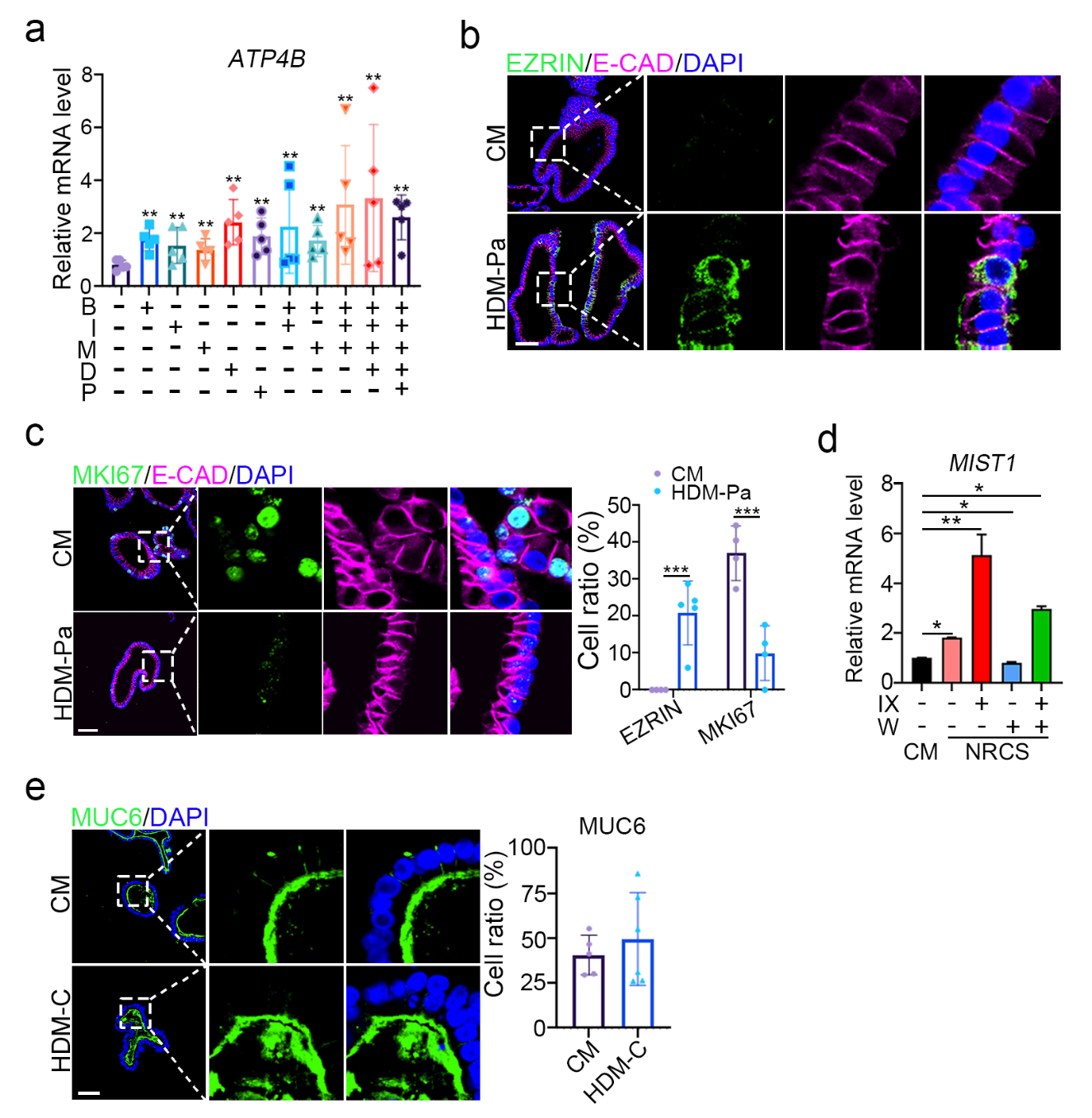
**

**Figure S14. Different requirements for differentiation of parietal and chief cells in human and mouse organoids, related to Figure 6.**

a, The mRNA expression of *ATP4B* in human gastric organoids cultured under the indicated conditions for 2 days. Statistical significance of each group in (a) was compared to the control group without BIMDP. B: BMP4, I: Isx-9, M: Metformin, D: DY131, P: PD0325901. b-c, Immunofluorescent staining of EZRIN, MKI67 and E-CAD of human gastric organoids, and quantification (b, n=5; c, n=4). Scale bars, 50 μm. d, The mRNA expression of *MIST1* in human gastric organoids cultured under the indicated conditions for 2 days. IX: IXA4, W: Wnt3A, NRCS: add Noggin, Rspondin1, CHIR-99021 and SB431542 in the basal culture medium. e, Immunofluorescent staining of MUC6 in human gastric organoids, and quantification (n=5). Scale bars, 50 μm. All immunofluorescence images were counter-stained with DAPI to show nuclei. Statistical significances were determined by unpaired multiple t test. *p < 0.05, **p < 0.01, ***p < 0.001.
